# The α- and β-Subunit Boundary at the Stem of the Mushroom-Like α_3_β_3_-Type Oxygenase Component of Rieske Non-Heme Iron Oxygenases Is the Rieske-Type Ferredoxin-Binding Site

**DOI:** 10.1128/aem.00835-22

**Published:** 2022-07-13

**Authors:** Pi-Cheng Tsai, Joydeep Chakraborty, Chiho Suzuki-Minakuchi, Tohru Terada, Tatsurou Kotake, Jun Matsuzawa, Kazunori Okada, Hideaki Nojiri

**Affiliations:** a Agro-Biotechnology Research Center, Graduate School of Agricultural and Life Sciences, The University of Tokyo, Tokyo, Japan; b Department of Microbiology, School of Science, RK University, Rajkot, Gujarat, India; c Collaborative Research Institute for Innovative Microbiology, The University of Tokyo, Tokyo, Japan; d Department of Biotechnology, Graduate School of Agricultural and Life Sciences, The University of Tokyo, Tokyo, Japan; Shanghai Jiao Tong University

**Keywords:** Rieske non-heme iron oxygenase, dioxygenases, electron transport, ferredoxin, protein-protein interactions

## Abstract

Cumene dioxygenase (CumDO) is an initial enzyme in the cumene degradation pathway of Pseudomonas fluorescens IP01 and is a Rieske non-heme iron oxygenase (RO) that comprises two electron transfer components (reductase [CumDO-R] and Rieske-type ferredoxin [CumDO-F]) and one catalytic component (α_3_β_3_-type oxygenase [CumDO-O]). Catalysis is triggered by electrons that are transferred from NAD(P)H to CumDO-O by CumDO-R and CumDO-F. To investigate the binding mode between CumDO-F and CumDO-O and to identify the key CumDO-O amino acid residues for binding, we simulated docking between the CumDO-O crystal structure and predicted model of CumDO-F and identified two potential binding sites: one is at the side-wise site and the other is at the top-wise site in mushroom-like CumDO-O. Then, we performed alanine mutagenesis of 16 surface amino acid residues at two potential binding sites. The results of reduction efficiency analyses using the purified components indicated that CumDO-F bound at the side-wise site of CumDO-O, and K117 of the α-subunit and R65 of the β-subunit were critical for the interaction. Moreover, these two positively charged residues are well conserved in α_3_β_3_-type oxygenase components of ROs whose electron donors are Rieske-type ferredoxins. Given that these residues were not conserved if the electron donors were different types of ferredoxins or reductases, the side-wise site of the mushroom-like structure is thought to be the common binding site between Rieske-type ferredoxin and α_3_β_3_-type oxygenase components in ROs.

**IMPORTANCE** We clarified the critical amino acid residues of the oxygenase component (Oxy) of Rieske non-heme iron oxygenase (RO) for binding with Rieske-type ferredoxin (Fd). Our results showed that Rieske-type Fd-binding site is commonly located at the stem (side-wise site) of the mushroom-like α_3_β_3_ quaternary structure in many ROs. The resultant binding site was totally different from those reported at the top-wise site of the doughnut-like α_3_-type Oxy, although α_3_-type Oxys correspond to the cap (α_3_ subunit part) of the mushroom-like α_3_β_3_-type Oxys. Critical amino acid residues detected in this study were not conserved if the electron donors of Oxys were different types of Fds or reductases. Altogether, we can suggest that unique binding modes between Oxys and electron donors have evolved, depending on the nature of the electron donors, despite Oxy molecules having shared α_3_β_3_ quaternary structures.

## INTRODUCTION

Aromatic compounds are used by some microorganisms as carbon and/or energy sources and are transformed into nonhazardous or less-hazardous substances (1). Bioremediation removes aromatic compounds from the environment using microorganisms and is safer and more economical than physical or chemical methods (2). Under aerobic conditions, microorganisms overcome the resonance stabilization energies of aromatic ring systems using oxygenases, which are oxidoreductases that add one (monooxygenases, also called hydroxylases) or two (dioxygenases) atoms of atmospheric oxygen to aromatic compounds to convert the aromatic ring (3–5).

Rieske non-heme iron oxygenases (ROs) are implicated in generation of *cis*-dihydrodiol metabolites at the initial step of degradation of various aromatic compounds, such as naphthalene, biphenyl, isopropylbenzene (cumene), and carbazole (6). ROs are composed of one or two electron transfer components (reductase [Red] alone or both Red and ferredoxin [Fd]) and a catalytic component (terminal oxygenase [Oxy]) (7). The reaction is triggered by electrons from one molecule of NAD(P)H, which are transferred directly or via an Fd from Red to the Oxy (6, 8) to dihydroxylate one molecule of substrate.

Red is the first component of the electron transfer chain of ROs. Red is a direct electron donor of Oxy in two-component ROs, which are classified in class I in Batie’s classification system (9). Three-component ROs have Fd as the second electron transfer component, which shuttles electrons between Red and Oxy by noncovalent binding. Three-component ROs are classified into class II or III based on the nature of their Red, and the class II ROs can be divided into class IIA or IIB based on the type of Fd (9). The Rieske cluster of the α-subunit of Oxy components receives electrons from the electron transfer component and transfers them to the mononuclear iron (active center) of the neighboring α-subunit. Most Oxy components also have an additional small β-subunit with no prosthetic group, the function of which has been suggested to be structural (10–15). All Oxy structures exhibit an α_3_ doughnut-like or α_3_β_3_ mushroom-like quaternary structure.

**FIG 1:**
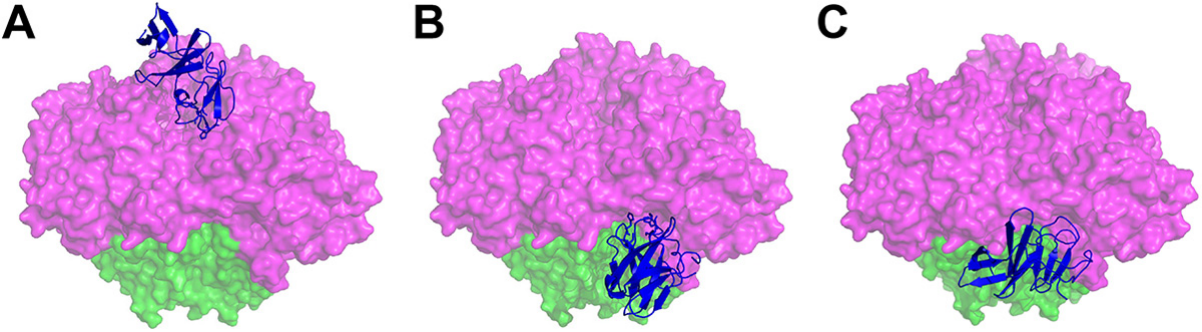
Potential binding sites of CumDO-F on CumDO-O. Two potential binding sites were predicted by docking simulations using GRAMM-X. In panels A and B, CumDO-F models based on the crystal structures of the TDO-F (PDB entry 4EMJ) (37) were used, while in panel C, the CumDO-F model prepared from BDO-F (PDB entry 1FQT) (8) was used. The surfaces of the α- and β-subunits of CumDO-O (PDB entry 1WQL) are shown in magenta and green, respectively. Homology-modeled CumDO-Fs are shown in blue in the ribbon model. (A) Binding at the α-subunit interface at the top-wise site and (B and C) binding at the α- and β-subunit boundary at the side-wise site are shown.

Electron transfer is essential for aromatic degradation by ROs and is related to catalytic activity. The binding site of Red and Fd has been determined based on the structure of the Red-Fd complex of biphenyl dioxygenase (BDO) from *Acidovorax* sp. strain KKS102 and the Red-Fd complex model of toluene dioxygenase (TDO) from Pseudomonas putida F1 (16, 17). The binding site between Fd and Oxy has been determined only for the Fd-Oxy complex of carbazole 1,9a-dioxygenase (CARDO), which has an α_3_-type Oxy (18). That is, Fd of CARDO (CARDO-F) bound in the hydrophobic groove surrounded by charged residues at the α-subunit interface of Oxy in the CARDO (CARDO-O) component. The interaction between Fd and α_3_β_3_-type Oxy has been predicted by docking simulations. Friemann et al. (17) and Khara et al. (19) performed docking simulations of Fd and the α_3_β_3_-type Oxy of TDO from P. putida F1 and aromatic hydrocarbon dioxygenase from *Sphingobium* sp. strain PNB. The results implied that Fd binds to Oxy at the interface of the α-subunit at the top-wise site (on the cap of the mushroom-like structure; similar position to Fig. 1A) or the interface of the α- and β-subunits at the side-wise site (at the stem of the mushroom-shaped structure; similar position to Fig. 1B and C). Kumari et al. (20) performed docking simulations of Fd and α_3_β_3_-type Oxy components of 3-nitrotoluene dioxygenase (3NTDO) from *Diaphorobacter* sp. strain DS2 and demonstrated that Fd binds at the α-subunit interface at the top-wise site (20). The putative binding site of the top-wise site roughly corresponds to that in the CARDO complex (18).

Cumene dioxygenase (CumDO; EC 1.14.12.−) from Pseudomonas fluorescens IP01, which is a three-component RO system, catalyzes the initial reaction of the cumene degradation pathway. *cumA1*, *cumA2*, *cumA3*, and *cumA4* genes, which encode the CumDO three-component oxygenase system comprising α- and β-subunits of Oxy, Fd, and Red, have been isolated (21). Electrons are transferred from NADH via flavin adenine dinucleotide (FAD) of the iron-sulfur flavoprotein Red (CumDO-R) and Rieske cluster of Rieske-type Fd (CumDO-F) to the Rieske cluster of α_3_β_3_-type Oxy (CumDO-O). CumDO-O has an α_3_β_3_ mushroom-like quaternary structure (22). CumDO is of class IIB according to Batie’s classification (9), as are BDO and TDO, which also have Rieske-type Fds and α_3_β_3_-type Oxys (17, 23–28). CumDO proteins possess 64 to 74%, 50 to 59%, 50 to 77%, and 48 to 73% identities with the α- and β-subunits of Oxy, Fd, and Red of BDO from Paraburkholderia xenovorans LB400 (formerly identified as Burkholderia xenovorans), BDO from Rhodococcus jostii RHA1, and TDO from P. putida F1, respectively (22). The structures of the α- and β-subunits of CumDO-O have root mean square deviation (RMSD) values of 0.94 to 1.45 Å for the full-length α-subunit and 0.89 to 1.43 Å for the full-length β-subunit of Oxy components of BDO and TDO, suggesting that CumDO-O is structurally similar to those of BDO and TDO (17, 22, 24, 29). Therefore, CumDO has potential as a model of the TDO/BDO subfamily (30, 31).

In the current study, we evaluated the mode of binding between Rieske-type Fd and α_3_β_3_-type Oxy using the CumDO system. Electron transfer activities from CumDO-F to alanine-substituted CumDO-O were determined to identify the residues essential for component interactions. In addition, we discuss the binding mode between Fd and Oxy in various ROs based on reported crystal structures and the conservation of important amino acid residues identified by CumDO analysis.

## RESULTS

### Prediction of potential binding sites of CumDO-F on CumDO-O.

The structure of CumDO-F has not been determined. The structure of CumDO-F was generated by homology modeling using SWISS-MODEL (32–36), based on the 2.4-Å structure of Fd of TDO (TDO-F [PDB entry 4EMJ]; 56% sequence identity to CumDO-F) (37) or the 1.6-Å structure of Fd of BDO (BDO-F [PDB entry 1FQT]; 78% sequence identity to CumDO-F) (8). The QMEAN numbers of TDO-F-based and BDO-F-based model structures were −1.33 and −0.87, respectively, indicating reliable model quality.

We performed docking simulations with CumDO-O and two homology-modeled CumDO-Fs (TDO-F-based and BDO-F-based models). In total, 20 each docking modes were generated by docking simulations. Electron transfer occurs when Rieske clusters are separated by <14 Å (38). In 17 simulations using the TDO-F-based model and 16 simulations using the BDO-F-based model, the two Rieske clusters were separated by ≥18 Å, preventing the transfer of electrons. Therefore, these binding modes were excluded. When the TDO-F-based model was used, one simulation result, in which CumDO-F bound at a side-wise site of CumDO-O (at the interface of the α- and β-subunits constituting the stem of the mushroom-like structure), showed an ~11-Å distance between two Rieske clusters. When the BDO-F-based model was used, in four simulation results, CumDO-F bound at a side-wise site of CumDO-O with a 14- to 16-Å distance between two Rieske clusters. On the other hand, in two simulation results using the TDO-F-based model, CumDO-F bound at a top-wise site of CumDO-O (at the interface of two α-subunits constituting the cap of the mushroom-like structure) showed an ~16-Å distance between two Rieske clusters. Although the observed distance was longer than the 14-Å threshold (38), this binding mode was similar to those of CARDO-F and CARDO-O (18). Moreover, one of the previous works found the best docking model with a 16.1-Å distance between two Rieske clusters (19). Thus, we considered that the proposed top-wise site may be the CumDO-F-binding position.

These two possible CumDO-F binding structures are shown in Fig. 1A (top-wise site binding) and 1BC (side-wise site binding). In both potential CumDO-F binding sites on CumDO-O, a hydrophobic groove was surrounded by several charged amino acid residues (data not shown). These features are similar to those of the CARDO-F-binding site on CARDO-O (18), although the amino acid residues constituting the potential binding sites were not conserved between the two Oxy molecules.

### Effect of alanine substitutions on electron transfer efficiency between CumDO-O and CumDO-F components.

**FIG 2:**
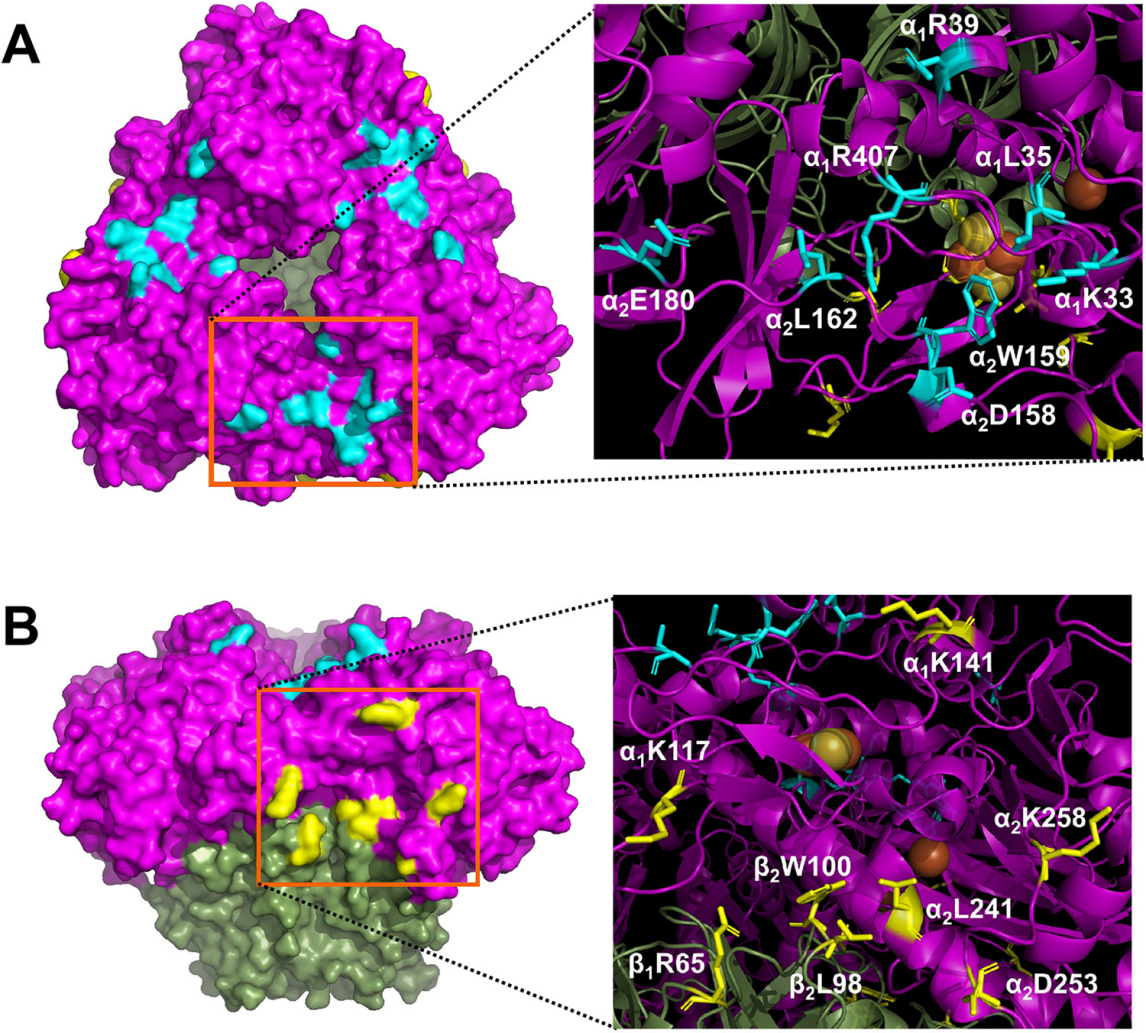
Amino acid residues of CumDO-O for alanine substitution. Each of 16 amino acid residues of CumDO-O (light blue and yellow) was replaced with alanine. In both panels, the left images show the molecular surface of CumDO-O, with the positions of the substituted residues and two potential binding sites indicated by orange squares, and the right images show ribbon models of potential binding sites, in which alanine-substituted residues are shown in sticks. Lys33, Leu35, Arg39, and Arg407 of α subunit 1 (α_1_ subunit) and Asp158, Trp159, Leu162, and Glu180 of neighboring α subunit 2 (α_2_ subunit) were located at the top-wise site (A). Lys117 and Lys141 of the α_1_ subunit, Leu241, Asp253, and Lys258 of the α_2_ subunit, Arg65 of β subunit 1 (β_1_ subunit), and Leu98 and Trp100 of the neighboring β subunit 2 (β_2_ subunit) were located at the side-wise site (B). α- and β-subunits of CumDO-O are shown in magenta and green, respectively.

Alanine substitutions were introduced to the surface amino acid residues of CumDO-O, which constitute two potential CumDO-F-binding surfaces. Sixteen charged or hydrophobic residues were chosen for alanine substitution (Fig. 2). Eight residues (Lys33, Leu35, Arg39, and Arg407 on α-subunit 1 [α_1_] and Asp158, Trp159, Leu162, and Glu180 on neighboring α-subunit 2 [α_2_]) were located at the top-wise site. Eight residues (Lys117 and Lys141 on α_1_, Leu241, Asp253, and Lys258 on α_2_, Arg65 on β-subunit 1 [β_1_], and Leu98 and Trp100 on β-subunit 2 [β_2_]) were located at the side-wise sites. Here, the single-alanine-substituted CumDO-Os are designated α_1_K33A, α_1_L35A, α_1_R39A, α_1_R407A, α_2_D158A, α_2_W159A, α_2_L162A, α_2_E180A, α_1_K117A, α_1_K141A, α_2_L241A, α_2_D253A, α_2_K258A, β_1_R65A, β_2_L98A, and β_2_W100A. CumDO-O and its derivatives were tagged with six histidine residues at the C terminus, considering that the C terminus of the α-subunit of CumDO-O was far away from the α- and β-subunit interface of Oxy and the active pocket of CumDO-O. CumDO-O and its derivatives contain 51-kDa α- and 21-kDa β-subunits and form a 150- to 200-kDa complex in solution (see Fig. S1 in the supplemental material). This indicates that CumDO-O and its derivatives are hetero-hexamer in solution. Also, to confirm that the secondary structure of CumDO-O was not disrupted by alanine substitution, circular dichroism (CD) measurement was performed. There was no significant difference in CD spectra (Fig. S2). Therefore, we can consider that His tag has no effect or a negligible effect on the activity and formation of the hetero-hexamer.

To evaluate the contribution of the residues described above to electron transfer between the Rieske clusters of CumDO-F and CumDO-O, the reduction efficiencies of 16 single-alanine-substituted CumDO-Os by wild-type (WT) CumDO-F were measured. When CumDO-F binding with CumDO-O, the electron from Rieske cluster of CumDO-F reduced the Rieske cluster of CumDO-O. The reduction of the Rieske cluster of CumDO-O could be measured by absorbance at a 457-nm wavelength (Fig. S3A).

**FIG 3:**
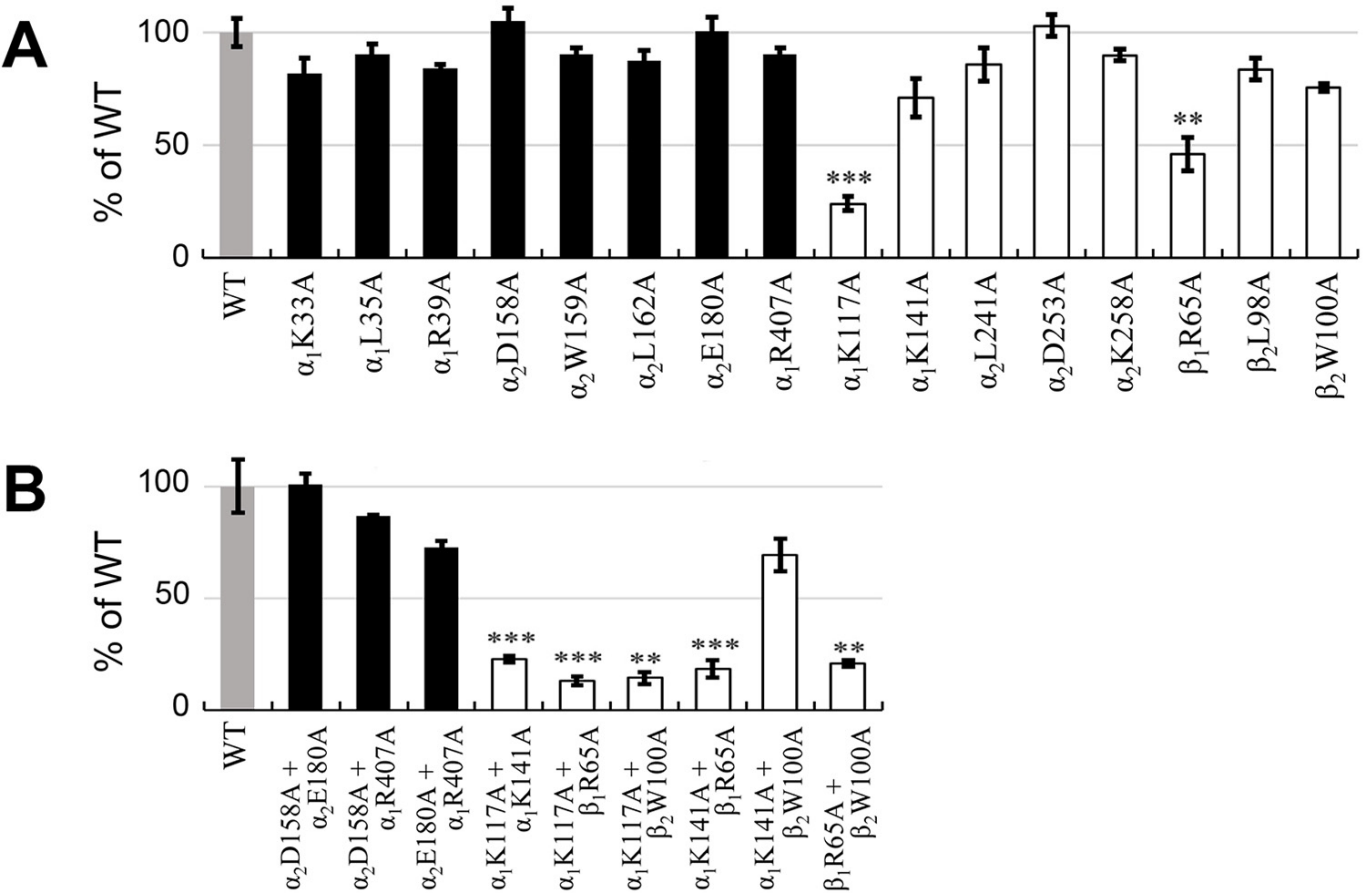
Reduction efficiencies of alanine-substituted CumDO-Os by CumDO-F. Panels A and B show the reduction efficiencies of single-alanine-substituted CumDO-Os and double-alanine-substituted CumDO-Os, respectively. Results for WT CumDO-O (set at 100%) are shown as gray bars. Black and white bars are the reduction efficiencies of CumDO-O derivatives with amino acid substitutions at the top-wise and side-wise potential CumDO-F-binding sites, respectively. Error bars indicate standard deviations from three independent experiments. The data were assessed using Student's *t* test with *P* values of <0.005 (**) or <0.001 (***) for alanine-substituted CumDO-Os compared to the WT.

All single-alanine substitutions at top-wise sites showed no significant decrease in reduction efficiencies compared with WT CumDO-O (Fig. 3A; Fig. S3). In contrast, the reduction efficiencies of some CumDO-O derivatives with alanine substitutions at side-wise sites were significantly decreased (Fig. 3A; Fig. S3). In particular, the efficiencies of α_1_K117A (Fig. S3K) and β_1_R65A (Fig. S3P) decreased to 21% ± 1.1% (*n* = 3) and 46% ± 3.0% (*n* = 3) compared to WT CumDO-O. The efficiencies of α_1_K141A and β_2_W100A decreased slightly but nonsignificantly (to 71 to 75%).

Based on the above findings, double-alanine-substituted CumDO-Os were constructed. A total of eight residues was selected: four (α_2_D158A, α_2_W159A, α_2_E180A, and α_1_R407A) corresponding to the top-wise site and four (α_1_K117A, α_1_K141A, β_1_R65A, and β_2_W100A) to the side-wise site. Of the 12 double-alanine-substituted CumDO-Os, 9 (α_2_D158A α_2_E180A, α_2_D158A α_1_R407A, α_2_E180A α_1_R407A, α_1_K117A α_1_K141A, α_1_K117A β_1_R65A, α_1_K117A β_2_W100A, α_1_K141A β_1_R65A, α_1_K141A β_2_W100A, and β_1_R65A β_2_W100A) were successfully expressed in Escherichia coli and purified as for WT CumDO-O. CD measurement confirmed that 9 double-alanine-substituted CumDO-Os showed similar CD spectra to the WT (Fig. S2), implying no change in secondary structure. Three double-alanine-substituted CumDO-O derivatives, α_2_D158A α_2_W159A, α_2_W159A α_2_E180A, and α_2_W159A α_2_R407A, could not be expressed in E. coli.

Three combinations of double-alanine substitutions at top-wise sites showed no significant decrease in reduction efficiencies compared with WT CumDO-O (Fig. 3B; Fig. S3S to U). In contrast, the reduction efficiencies of double-alanine substituents, which included α_1_K117A or β_1_R65A, were significantly decreased to <20%, while that of α_1_K141A β_2_W100A was decreased 69% ± 2.2% (*n* = 3) (Fig. 3B; Fig. S3V-AA). These results indicate that the interaction between CumDO-F and CumDO-O is disrupted significantly when α_1_K117 or β_1_R65 was substituted for with alanine. This means CumDO-F binds at the side-wise site of CumDO-O and transfers electrons to the Rieske cluster of CumDO-O.

### α_1_K117 and β_1_R65 were conserved in most α_3_β_3_-type Oxy components of ROs.

**FIG 4:**
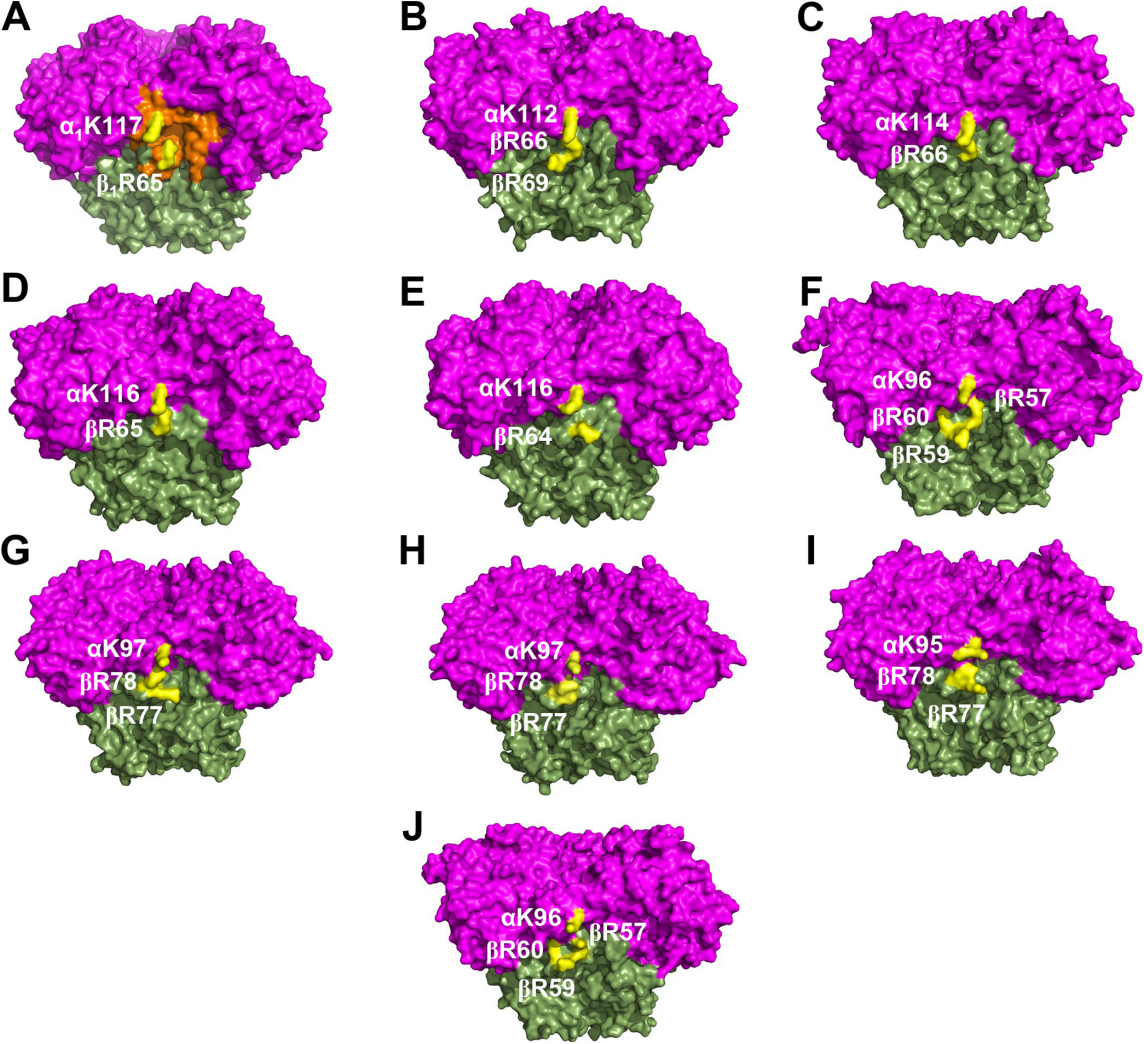
Conserved charged residues in the structures of α_3_β_3_-type Oxy components. Structures of different α_3_β_3_-type Oxys of 10 RO systems are shown: (A) CumDO from P. fluorescens IP01 (PDB entry 1WQL), (B) TDO from P. putida F1 (PDB entry 3EN1), (C) BDO from *R. jostii* RHA1 (PDB entry 1ULI), (D) BDO from *P. pnomenusa* B-356 (PDB entry 3GZY), (E) BDO from *B. xenovorans* LB400 (PDB entry 2XR8), (F) BDO from Sphingomonas yanoikuyae B1 (PDB entry 2GBX), (G) NDO from Pseudomonas sp. strain NCIB9816-4 (PDB entry 1NDO), (H) NDO from Pseudomonas sp. strain C18 (PDB entry 4HJL), (I) NBDO from *Comamonas* sp. strain JS765 (PDB entry 2BMO), and (J) PAH-hydroxylating dioxygenase from *Sphingomonas* sp. strain CHY-1 (PDB entry 2CKF) are shown in the side view. α- and β-subunits are shown in magenta and green, respectively. The groove of the potential CumDO-F binding site is shown in orange in panel A. Conserved positive-charged residues are in yellow.

**FIG 5:**
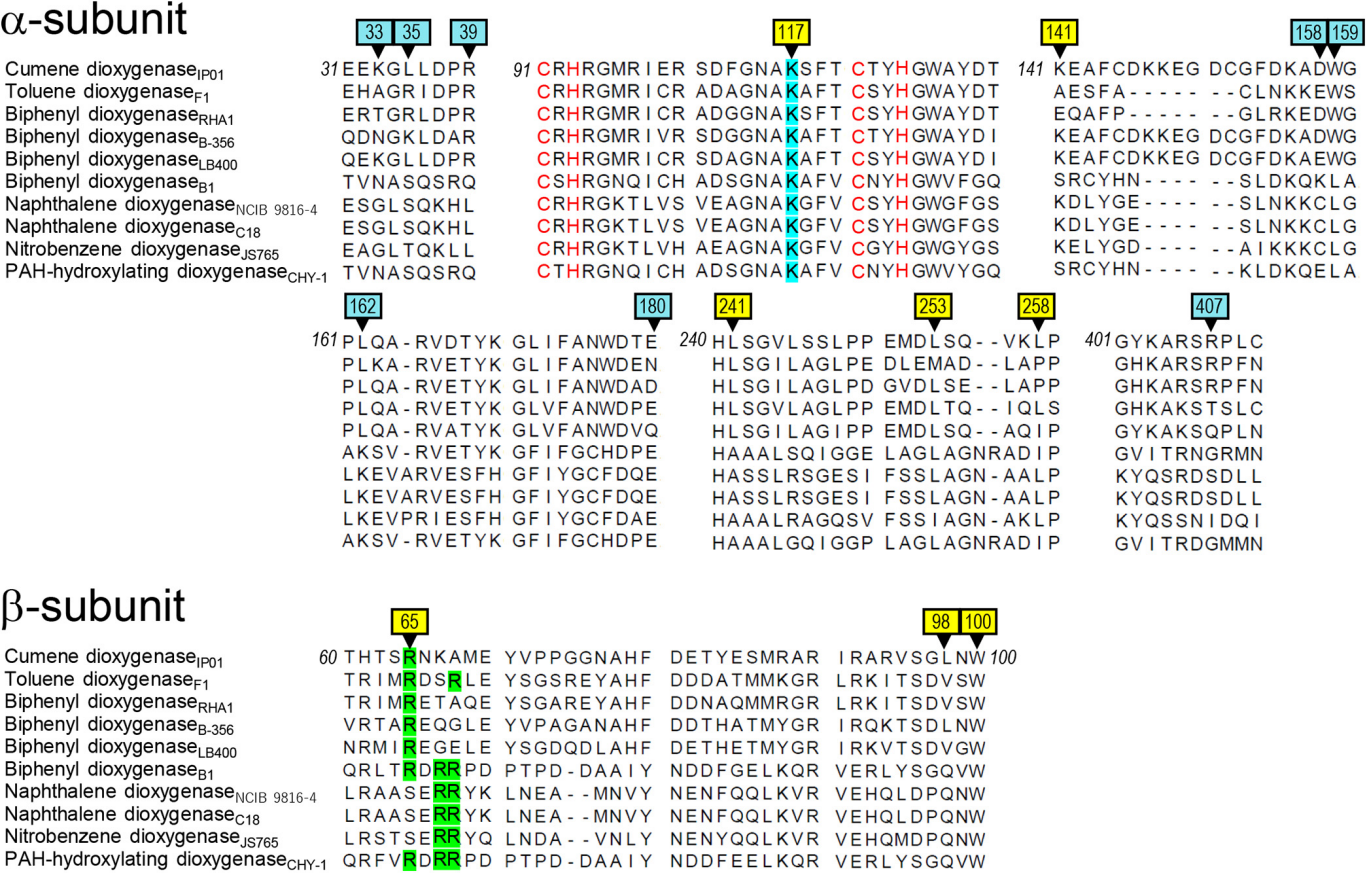
Amino acid sequence alignments of α- and β-subunits of 10 structure-solved Oxys. Parts of amino acid sequence alignments of Oxy components of 10 RO systems are shown. Enzyme names followed by origins (subscripted) are shown at left. Alanine-substituted residues (arrowheads) at the top-wise and side-wise sides are shown by light blue and yellow backgrounds, respectively. Conserved positive amino acid residues in the side-wise putative Fd-binding site (Fig. 4) are shown in light blue and light green in the α- and β-subunit sequences, respectively. Cys and His residues in the α-subunit sequences involved in the coordination of Rieske clusters are shown in red. Numbers at termini show the positions of terminal amino acid residues in CumDO-O protein.

The crystal structures of α_3_β_3_-type Oxy components of 10 ROs have been reported: CumDO-O from P. fluorescens IP01 (PDB entry 1WQL) (22), TDO-O from P. putida F1 (PDB entry 3EN1) (17), BDO-O from *R. jostii* RHA1 (PDB entry 1ULI) (24), BDO-O from Pandoraea pnomenusa B-356 (PDB entry 3GZY) (39), BDO-O from *B. xenovorans* LB400 (PDB entry 2XR8) (29), BDO-O from Sphingobium yanoikuyae B1 (PDB entry 2GBX) (40), NDO-O from Pseudomonas sp. strain NCIB9816-4 (PDB entry 1NDO) (11), NDO-O from Pseudomonas sp. strain C18 (PDB entry 4HJL), Oxy of nitrobenzene dioxygenase (NBDO-O) from *Comamonas* sp. strain JS765 (PDB entry 2BMO) (41), and Oxy of PAH-hydroxylation dioxygenase from *Sphingomonas* sp. strain CHY-1 (PDB entry 2CKF) (42). These 10 ROs have a Rieske-type Fd as electron donors for the Oxy components. The Oxy structures were superimposed by overlapping Rieske clusters and mononuclear irons, and we compared the loci corresponding to the side-wise site at the α- and β-subunit boundary, especially positively charged residues. The α- and β-subunit boundary formed a groove in CumDO-O (Fig. 4A, orange). K117 of the α-subunit and R65 of the β-subunit were at the edge of the groove. We found similar grooves in all Oxy structures, and the positions of the positively charged residues were conserved in all Oxy components (Fig. 4B to J). An amino acid sequence alignment was performed for the α- and β-subunits of oxygenase components of the 10 ROs. Lys residues corresponding to α_1_K117 in CumDO-O were conserved in all α_3_β_3_-type Oxy components (Fig. 5, light blue). Although Arg residues corresponding to β_1_R65 of CumDO-O were not conserved in the β subunits of three ROs, there were two conserved Arg residues at +2 and +3 from those corresponding to β_1_Arg65 in the β-subunits of five ROs, including the three described above (Fig. 5, light green). The positions of two (or three) Arg residues were shared with those of Arg residues, including β_1_Arg65, and all Arg residues were situated at the edge of the groove (Fig. 4). Five ROs with the above two (or three) Arg residues in the Oxy β-subunits are Batie’s class III (9), unlike CumDO (class IIB). Also, the charged or hydrophobic amino acid residues which we substituted for alanine (Fig. 5, black arrowheads) were likely to be more conserved at side-wise sites than at top-wise sites.

**FIG 6:**
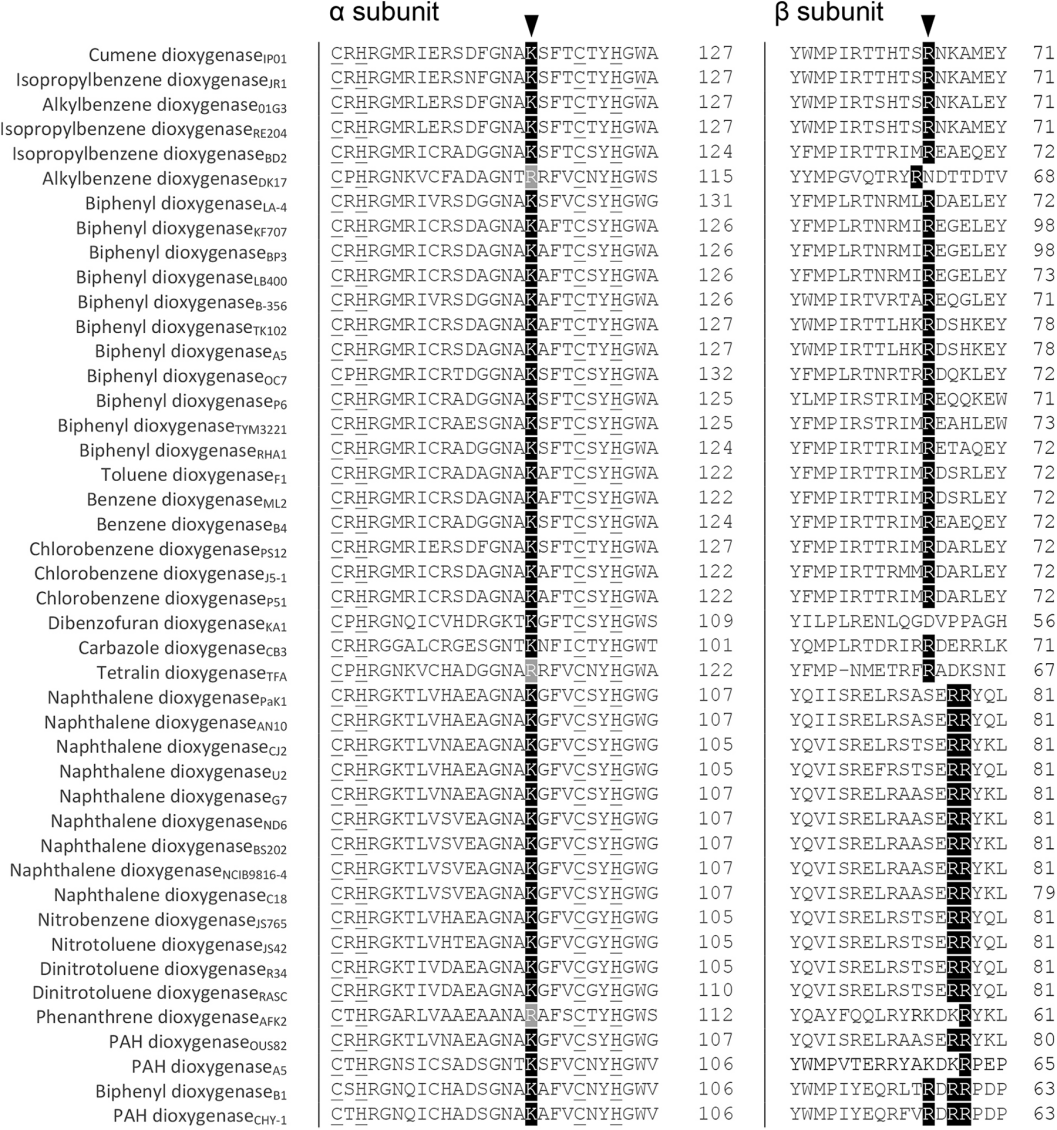
Amino acid sequence alignments of α- and β-subunits of CumDO Oxy with those of α_3_β_3_-type Oxys coupled with Rieske-type Fds. Only the parts of Oxy including the potential important amino acid residues for binding to Rieske-type Fds are shown. Conserved and similar amino acid residues at positions crucial for binding between α_3_β_3_-type Oxys and Rieske-type Fds (indicated by arrowheads) are darkly and lightly shaded, respectively. Four ligands for the Rieske cluster in α subunits are underlined. Numbers at right show the positions of terminal amino acid residues. Enzyme names followed by origins (subscripted) are shown at left.

Next, we assessed whether the Lys residue in the α subunit and Arg residue(s) in the β subunit are conserved in α_3_β_3_-type Oxy components in RO systems. First, we used 44 representative ROs coupled to Rieske-type Fds as electron donors (Batie’s class IIB and III) (9) (Table S1). Figure 6 shows alignments of the corresponding regions of the α- and β-subunits of CumDO and selected Oxys. Lys residues are conserved in α subunits but replaced by Arg in RO Oxys for alkylbenzene (*Rhodococcus* sp. strain DK17), tetralin (Sphingopyxis macrogoltabida TFA), and phenanthrene (Alcaligenes faecalis AFK2) (Fig. 6, left). In β-subunits, one to three Arg residues are conserved in all Oxy components, excluding that of dibenzofuran dioxygenase from *Novosphingobium* sp. strain KA1 (Fig. 6, right). The number of Arg residues is conserved in RO, depending on the Batie’s classification (9): one Arg is conserved in class IIB ROs, and two Arg residues at +2 and +3 are conserved in class III ROs. These Lys and Arg residues are not conserved in α_3_β_3_-type Oxy components whose electron donor proteins are different types of Fd (Fig. S4A) or Red (Fig. S4B) proteins. These results suggest that, when Rieske-type Fd is an electron donor, α_3_β_3_-type Oxy components share an Fd-binding mode, in which Fd molecules bind to the α- and β-subunit boundary region at the stem (side-wise site) of mushroom-like Oxy structures. In addition, Lys residues in α subunits and Arg residues in β subunits are involved in the specific binding between α_3_β_3_-type Oxy and Rieske-type Fd.

### Conserved residues on the surfaces of Rieske-type Fds.

**FIG 7:**
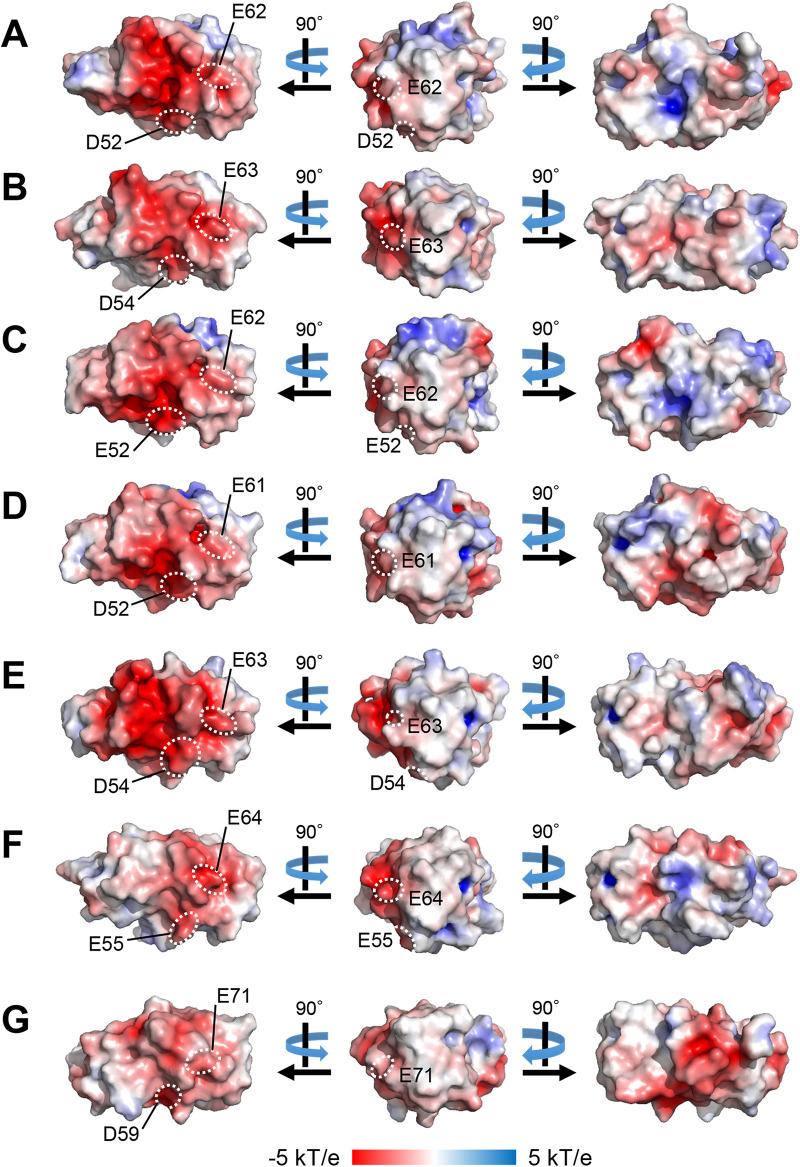
Surface electrostatic potential of seven structure-solved Rieske-type Fds. Surface electrostatic potential of (A) BDO-F from *B. xenovorans* LB400 (PDB entry 1FQT), (B) BDO-F from *B. xenovorans* B1 (PDB entry 2I7F), (C) BDO-F from *Acidovorax* sp. strain KKS102 (PDB entry 2E4P), (D) TDO-F from P. putida F1 (PDB entry 3DQY), (E) NDO-F from Pseudomonas sp. strain NCIB9816-4 (PDB entry 2QPZ), (F) CARDO-F from *P. resinovorans* CA10 (PDB entry 1VCK), and (G) CARDO-F from Nocardioides aromaticivorans IC177 (PDB entry 3GCE) are shown. Fds in panels A to E transfer electrons to α_3_β_3_-type Oxys, and Fds in panels F and G transfer electrons to α_3_-type Oxys. Fd molecules in the middle are facing the binding surfaces of Oxy molecules, and Rieske clusters are located at their tips. Positive and negative potential regions are shown in blue and red, respectively. Positions of conserved Asp/Glu residues are also shown.

**FIG 8:**
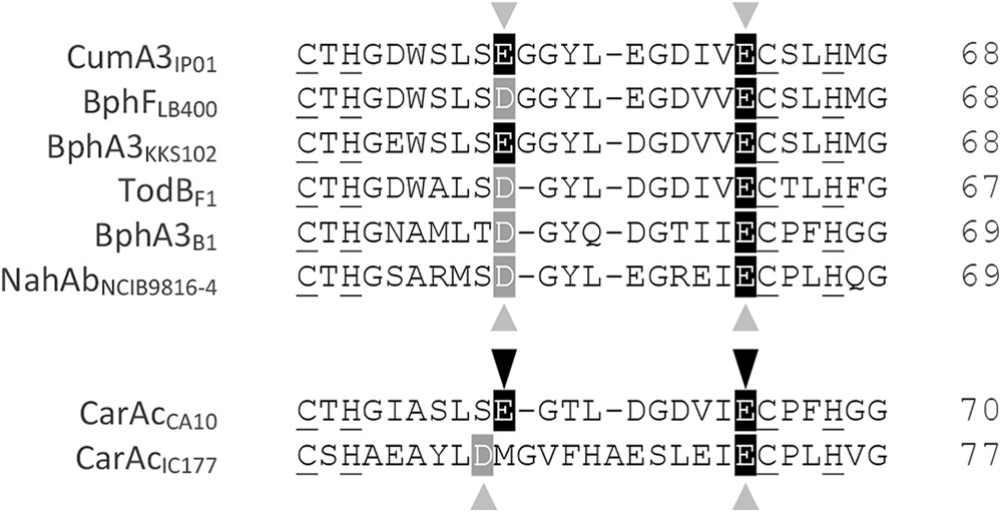
Amino acid sequence alignment of structure-solved Rieske-type Fds. The positions of two negatively charged Glu residues (Glu55 and Glu64), reported to mediate electrostatic interactions with positively charged amino acid residues of Oxy in the Oxy-Fd complex crystal of CARDO (18) are shown by black arrowheads and the corresponding Asp/Glu residues in other Fds by gray arrowheads. Amino acid residues identical or similar to CumDO-F (CumA3 protein) in other Fds are shaded in black and gray, respectively. The four conserved ligands for the Rieske cluster are underlined. Numbers at right show the positions of terminal amino acid residues. Protein names of Fds and their origins (subscripted) are shown at left.

We conducted multiple alignments of the Rieske-type Fds involved in RO systems. To date, the crystal structures of seven Rieske-type Fds have been reported. The counterparts of five Fds—BDO-F from *B. xenovorans* LB400 (PDB entry 1FQT) (8), BDO-F from S. yanoikuyae B1 (PDB entry 2I7F) (40), BDO-F from *Acidovorax* sp. strain KKS102 (PDB entry 2E4P) (16), TDO-F from P. putida F1 (PDB entry 3DQY) (17), and NDO-F from Pseudomonas sp. strain NCIB9816-4 (PDB entry 2QPZ) (43)—are α_3_β_3_-type Oxys. In contrast, those of CARDO-F from Pseudomonas resinovorans CA10 (PDB entry 1VCK) (44) and CARDO-F from Nocardioides aromaticivorans IC177 (PDB entry 3GCE) (45) are α_3_-type Oxys. Their crystal structures and surface electrostatic potentials are shown in Fig. 7. Images in the middle are facing the binding surfaces of Oxy molecules, and the Rieske clusters in Fd molecules are located close to the viewpoints. The molecular surface of Rieske cluster-surrounding regions was negatively charged, and the positions of negatively charged residues were conserved in all Rieske-type Fds (Fig. 7, left). Amino acid sequence alignment of the regions between the ligands for the Rieske cluster of the seven Fds and CumDO-F (CumA3 protein) showed that many amino acid residues are conserved (Fig. 8), and the amino acid sequences of Fds with α_3_β_3_-type Oxy electron receptors are likely to be more conserved. Electrostatic interactions are reportedly important for the interaction between the Oxy and Fd components of class III CARDO (18, 45). Based on the complex structure of CARDO-O and CARDO-F, the critical negatively charged residues on CARDO-F were determined to be Glu55 and Glu64 (18) (Fig. 7F). Negatively charged Asp/Glu residues were conserved in Fds, including CumDO-F (Fig. 8), and were at identical positions in all Fd molecules compared (Fig. 7). These data suggest that the conserved Asp/Glu residues are involved in Oxy-Fd binding and that their binding mode is shared by ROs.

### Proposed electron transfer pathway from CumDO-F to CumDO-O.

**FIG 9:**
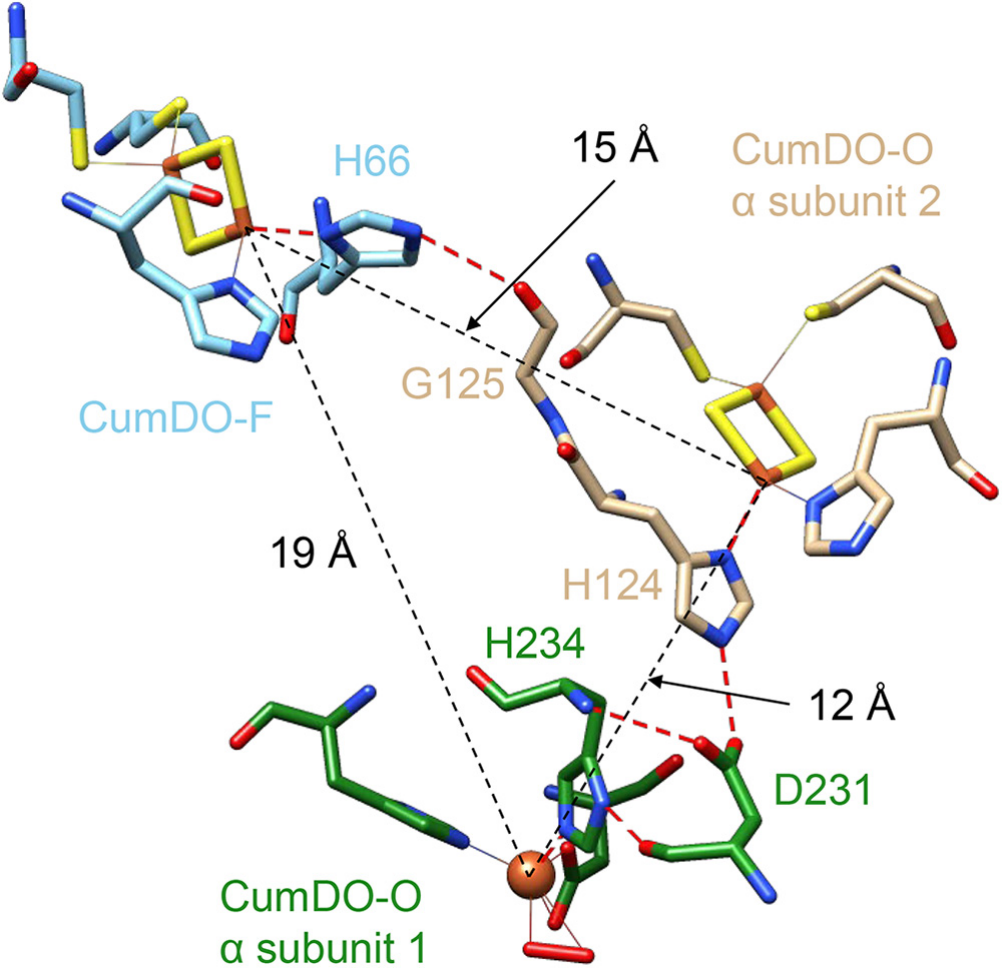
Possible electron transfer pathway from the Rieske cluster of CumDO-F to the non-heme iron of CumDO-O. The hydrogen bonds that mediate the electron transfer are indicated by red dashed lines. The atoms involved in the electron transfer and in the coordination with the iron ions are shown in a stick model. Nitrogen, oxygen, sulfur, and iron atoms are colored blue, red, yellow, and orange, respectively. Carbon atoms in CumDO-F are colored cyan, and those in α-subunits 1 and 2 of CumDO-O are colored green and brown, respectively. Distances among three electron transfer centers, two Rieske clusters, and an active site iron are shown by black dotted lines.

Using a CumDO-F model structure, a CumDO-F-binding structure at the side-wise site of CumDO-O was modeled. Based on the modeled structure, we inferred a possible electron transfer pathway between two Rieske clusters (Fig. 9). In the binding structure model, the distance between the two Rieske clusters was 15 Å, which is shorter than that between the CumDO-F Rieske cluster and active site iron (19 Å). The Nε2 atom of His66, a ligand of the Rieske cluster of CumDO-F, formed a hydrogen bond with the main-chain oxygen of Gly125, which is adjacent to His124, a ligand of the Rieske cluster of CumDO-O. The electron is therefore thought to be transferred through the route His66-Gly125-His124 (Fig. 9). Then, the electron is thought to be transferred from the CumDO-O Rieske cluster to the active site iron at the neighboring subunit via His124, Asn231, and His234. (The distance was 12 Å).

## DISCUSSION

Using CumDO as a model RO system containing α_3_β_3_-type Oxy, we showed that Rieske-type Fd binds at the α- and β-subunit boundary of Oxy, which corresponds to the side-wise site of the mushroom-like Oxy molecule (Fig. 4A). The conclusion is based on the amino acid replacement analyses, followed by the amino acid sequence comparison with other related enzymes. Unfortunately, the direct elucidation by structural analysis of the complex structure of CumDO-O and CumDO-F has not been successful, but we could propose the common binding manner of Rieske-type Fd to α_3_β_3_-type Oxy in ROs. Based on the docking simulation, we can propose the electron transfer pathway between the CumDO-F and CumDO-O (Fig. 9).

Before the first crystal structure of the Oxy component of RO was reported in 1998 (11), there was debate over the function of the β-subunit of α_3_β_3_-type Oxys of ROs. The β-subunit of α_3_β_3_-type Oxys of BDO from Pseudomonas pseudoalcaligenes KF707, 2,4-dinitrotoluene dioxygenase from *Burkholderia* sp. strain DNT, 2-nitrotoluene 2,3-dioxygenase from Pseudomonas sp. strain JS42, chlorobenzene dioxygenase from P. putida F1, and NDO from Pseudomonas sp. strain NCIB9816-4 did not influence substrate specificity and activity (10–14). However, the β-subunit in α_3_β_3_-type Oxys of toluate 1,2-dioxygenase from P. putida and BDO from Pandoraea pnomenusa B-356 was reported to affect their substrate specificity (46, 47). In these studies, oxygenation activities for the original substrates were determined in the reconstituted RO systems, which have an Oxy component consisting of the original α-subunits and β-subunits of different origins. If the degradation rate was decreased by replacement, the β-subunit was considered to be a determinant of substrate specificity. In fact, the β-subunit of the α_3_β_3_-type Oxy of TDO from P. putida F1 was essential for activity (48). The crystal structures of α_3_β_3_-type Oxy components indicate that β-subunits are far from the substrate-binding pockets (6), suggesting a structural role. This hypothesis is supported by the presence of α_3_-type Oxy in CARDO (15) and 2-oxoquinoline 8-monooxygenase (49). However, when Arg65 of the β-subunit of CumDO-O was substituted for with alanine, the Rieske cluster of Oxy was not significantly reduced by Fd. Given that the important arginine residues were conserved in the β-subunits of most α_3_β_3_-type Oxys (Fig. 5 and 6), the β-subunit of α_3_β_3_-type Oxys is implicated in Fd recognition and stabilization of Oxy-Fd complex formation.

The α_1_K117 and β_1_R65 residues played critical roles in the interaction between CumDO-F and CumDO-O and are conserved in most α_3_β_3_-type Oxys (Fig. 5 and 6). Also, their positions are conserved on the surfaces of structure-solved α_3_β_3_-type Oxys (Fig. 4). Therefore, the Fd-binding mode is likely to be conserved in α_3_β_3_-type Oxys and Rieske-type Fds in Batie’s class IIB and III ROs (9). Some three-component RO systems include non-Rieske-type Fds, like [3Fe-4S] or [4Fe-4S] ferredoxin and rubredoxin (31). Class I ROs lack a ferredoxin, and their Oxys receive electron directly from Red components (9). Interestingly, most Oxys coupled with [3Fe-4S] or [4Fe-4S] Fd, rubredoxin, and directly with Reds lacking Lys and Arg residues (see Fig. S4 in the supplemental material). These data indicated the evolution of a unique binding mode between Oxy and the electron donor, depending on the nature of the electron donors, despite Oxy molecules having shared α_3_β_3_ quaternary structures.

Surface Asp/Glu residues of the Rieske-type Fd CARDO-F are important for binding to the α_3_-type Oxy CARDO-O (18). Given that these Asp/Glu residues are conserved in Fds, they are likely to be important in Oxy-Fd binding in each system. The interaction between these Asp/Glu residues of Fds and Oxy surface residues (including Lys/Arg residues) needs to be investigated. In addition, the effects of amino acid replacements at α_1_K117 and β_1_R65 residues on the physical binding to Fd should be quantified in future, but our data may suggest that the binding mode between Rieske-type Fds and Oxys is shared by ROs, irrespective of the quaternary structures of their Oxy components. This may be a good example of convergent evolution of RO Oxy molecules. Interestingly, besides ROs, similar networks of negatively charged residues on the ferredoxins and positively charged residues on the oxygenases have also been reported with cytochrome P450 enzymes and their ferredoxins (50, 51).

## MATERIALS AND METHODS

### Bacterial strains, plasmids, and culture media.

E. coli strains BL21(DE3) (Novagen, WI) or JM109(DE3) (Toyobo, Osaka, Japan) harboring plasmid pET-26b(+) (Novagen), pUC118 (TaKaRa Bio, Inc., Shiga, Japan), or their derivatives were cultivated in Luria-Bertani (LB) medium (52) and SB medium (53).

### Construction of plasmids.

The C terminus of α-subunit of CumDO-O (CumA1) and N termini of CumDO-F (CumA3) and CumDO-R (CumA4) were tagged with six histidine residues. The β-subunit of CumDO-O (CumA2) was expressed as nontagged protein. An artificial XbaI site, stop codon, Shine-Dalgarno (SD) sequence, NdeI (5′-end), and SalI (3′-end) sites were created at the termini of *cumA1*-containing DNA fragments using the primer set in Table 1 and plasmid pIP103 (21) as the PCR template. A SalI site, stop codon, SD sequence, and NdeI (5′-end) and HindIII (3′-end) sites were created at the termini of *cumA2*-containing DNA fragment using the primer set in Table 1 and pIP103. PCR amplicons containing *cumA1* or *cumA2* were inserted into the XbaI and HindIII sites of pUC118, resulting in pUCumA1CA2. An XbaI site, stop codon, SD sequence, and NdeI (5′-end) and SalI (3′-end) sites were created at the termini of *cumA3*-containing DNA fragments using the primers in Table 1 and pIP103. The PCR amplicon was inserted into XbaI and SalI sites of pET-26b(+), resulting in pETCumA3N. An XbaI site, stop codon, SD sequence, and NdeI (5′-end) and SalI (3′-end) sites were created at the termini of *cumA4*-containing DNA fragments using the primers in Table 1 and pIP103. The PCR amplicon was inserted into XbaI and SalI sites of pUC118, resulting in pUCumA4N.

**TABLE 1:** Primers used in this study

Primer function	Direction^*a*^	Sequence
Cloning of CumDO component gene^*b*^		
*cumA1*	F	5′-GGG*TCTAGA*TAAGAAGGAGATATACATATGAGTTCAATAATAAATAAAGAAGTGCAGGAAG-3′
	R	5′-AAA*GTCGAC*TCAGTGGTGGTGGTGGTGGTGAGACTTTAGCGTGTCCCAACTCG-3′
*cumA2*	F	5′-AAA*GTCGAC*TAAGAAGGAGATATACATATGACATCCGCTGATTTGACAAAAC-3′
	R	5′-GGG*AAGCTT*AGAAAAACTGGCTGAGATTATTCGCTG-3′
*cumA3*	F	5-AAA*GAATTC*TAAGAAGGAGATATACATATGCACCACCACCACCACCACACTTTTTCCAAAGTTTGTGAAGTATCTGATG-3′
	R	5′-AAA*GTCGAC*TCATGGCGCTAGATACCCGG-3′
*cumA4*	F	5′-AAA*TCTAGA*TAAGAAGGAGATATACATATGCACCACCACCACCACCACATTAAATCAATCGTCATTATTGGTGCTGGC-3′
	R	5′-AAA*GTCGAC*TCACTCGCATCGCTCAGCTTTAG-3′
Alanine substitutions^*c*^		
α_1_K33A	F	5′-GATGAGGAAGCGGGGTTGCTTGATCC-3′
	R	5′-GGATCAAGCAACCCCGCTTCCTCATC-3′
α_1_L35A	F	5′-GGAAAAGGGGGCGCTTGATCCAC-3′
	R	5′-GTGGATCAAGCGCCCCCTTTTCC-3′
α_1_R39A	F	5′-GCTTGATCCAGCGATTTTCTCTGATCAGG-3′
	R	5′-CCTGATCAGAGAAAATCGCTGGATCAAGC-3′
α_1_K117A	F	5′-GGCAACGCAGCGTCATTTACCTGC-3′
	R	5′-GCAGGTAAATGACGCTGCGTTGCC-3′
α_1_K141A	F	5′-CCCTACGAGGCGGAGGCTTTTTGTG-3′
	R	5′-CACAAAAAGCCTCCGCCTCGTAGGG-3′
α_2_D158A	F	5′-CGACAAGGCCGCGTGGGGGCCGC-3′
	R	5′-GCGGCCCCCACGCGGCCTTGTCG-3′
α_2_W159A	F	5′-AAGGCCGACGCGGGGCCGCTGC-3′
	R	5′-GCAGCGGCCCCGCGTCGGCCTT-3′
α_2_L162A	F	5′-GGGGGCCGGCGCAAGCGCGGGTG-3′
	R	5′-CACCCGCGCTTGCGCCGGCCCCC-3′
α_2_E180A	F	5′-CTGGGATACCGCGGCCCCTGATTTG-3′
	R	5′-CAAATCAGGGGCCGCGGTATCCCAG-3′
α_2_L241A	F	5′-GATGGCGCATGCGTCAGGTGTATTGTCC-3′
	R	5′-GGACAATACACCTGACGCATGCGCCATC-3′
α_2_D253A	F	5′-GCCTGAAATGGCGTTGTCCCAAG-3′
	R	5′-CTTGGGACAACGCCATTTCAGGC-3′
α_2_K258A	F	5′-GTCCCAAGTAGCGTTACCGTCAAGTGGG-3′
	R	5′-CCCACTTGACGGTAACGCTACTTGGGAC-3′
α_1_R407A	F	5′-GGCTAGAAGTGCGCCTCTTTGTGCCC-3′
	R	5′-GGGCACAAAGAGGCGCACTTCTAGCC-3′
β_1_R65A	F	5′-CTCATACATCCGCGAATAAGGCGATG-3′
	R	5′-CATCGCCTTATTCGCGGATGTATGAG-3′
β_2_L98A	F	5′-GGTTTCGGGGGCGAACTGGACTGAAG-3′
	R	5′-CTTCAGTCCAGTTCGCCCCCGAAACC-3′
β_2_W100A	F	5′-CGGGGCTTAACGCGACTGAAGATCC-3′
	R	5′-GGATCTTCAGTCGCGTTAAGCCCCG-3′

a F, forward; R, reverse.

b Nucleotide sequence for restriction enzyme sites are italicized, and those for the His tag are underlined.

c The alanine codons introduced are underlined.

### Expression and purification of cumene dioxygenase components.

CumDO-R, CumDO-O, and alanine-substituted CumDO-O were expressed in E. coli JM109(DE3) cells transformed with pUCumA4N, pUCumA1CA2, or derivative plasmids. For expression of CumDO-F, the plasmid pETCumA3N and E. coli BL21(DE3) were used.

E. coli strains were grown in 15 mL of LB medium with 50 μg/mL kanamycin [for BL21(DE3)(pETCumA3N)] or 100 μg/mL ampicillin [for JM109(DE3)(pUCumA4N) or JM109(DE3)(pUCumA1CA2)] at 310 K for 6 h. Next, the 15-mL seed cultures were inoculated into 1.5 L of SB medium in a jar fermenter (B. E. Marubishi Co., Ltd., Tokyo, Japan) and incubated at 310 K for 2.5 h. Isopropyl-β-d-thiogalactopyranoside (IPTG) was added to final concentrations of 1.0, 0.1, and 0.5 mM to induce expression of CumDO-R, CumDO-F, and CumDO-O (including amino-acid-substituted derivatives), respectively. Induction by IPTG was continued for 12 h at 298 K for CumDO-R and CumDO-F and 303 K for CumDO-O. E. coli cells were harvested by centrifugation at 5,000 × *g* for 10 min, washed once with Hitrap A buffer (20 mM Tris-HCl, 0.5 M NaCl, and 10% glycerol [pH 7.4]), and resuspended in Hitrap A buffer. Crude cell extract was prepared by sonication for 10 min, followed by centrifugation at 25,000 × *g* for 1 h. The proteins were subjected to metal chelation chromatography (HiTrap chelating HP column; column volume, 5 mL) (Amersham Biosciences, NJ, USA) using a fast-protein liquid chromatography instrument (ÄKTA FPLC) (GE Healthcare Japan, Tokyo, Japan) according to the manufacturer’s recommendations. CumDO-R, CumDO-F, and CumDO-O were eluted with 150, 150, and 180 mM imidazole in Hitrap B buffer (20 mM Tris-HCl, 0.5 M NaCl, 10% glycerol, and 300 mM imidazole [pH 7.4]). The CumDO-O- and CumDO-R-containing fractions were pooled and concentrated using the Vivaspin 20 system (molecular cutoff, 10 kDa) (Sartorius, Goettingen, Germany), and CumDO-F was pooled and concentrated using the Vivaspin Turbo 15 system (molecular cutoff, 5 kDa) (Sartorius). The preparations were further purified by gel filtration chromatography (GFC) on a HiLoad_26/60 Superdex200 prep-grade column (GE Healthcare Japan, Tokyo, Japan) (for CumDO-R and CumDO-O) or HiLoad_26/60 Superdex75 prep-grade column (GE Healthcare Japan, Tokyo, Japan) (for CumDO-F) with GFC buffer (20 mM Tris-HCl, 0.2 M NaCl, and 10% glycerol [pH 7.4]). Protein-containing fractions were pooled and concentrated as described above. Protein concentrations were estimated by Bradford assay (Bio-Rad Laboratories, CA). The CumDO component solutions were frozen in liquid nitrogen and stored at 193 K until use.

### Site-directed mutagenesis.

Thirteen residues of the α-subunit (K33, L35, R39, K117, K141, D158, W159, L162, E180, L241, D253, K258, and R407) and three of the β-subunit (R65, L98, and W100) were substituted for with alanine, respectively. Mutations for single-alanine substitutions were introduced using the KOD-Plus-Mutagenesis kit (Toyobo, Osaka, Japan) for pUCumA1CA2. The resulting expression vectors were named pUCumA1CK33AA2 and pUCumA1CA2R65A. (The underlined parts were changed by the replacement introduced.) The nucleotide sequences of the primers used for mutagenesis are provided in Table 1.

Gene fragments encoding doubly substituted alanine-replaced CumDO-Os (α_2_D158A α_2_E180A, α_2_D158A α_2_R407A, α_2_E180A α_1_R407A, α_1_K117A α_1_K141A, and β_1_R65A β_2_W100A) were artificially synthesized as inserts of cloning vector pUC57-Kan by Genewiz Japan (Saitama, Japan). Artificial XbaI and SalI restriction sites were added at the 5′ and 3′ termini of *cumA1* fragments encoding the α-subunit, respectively. As for *cumA2* encoding the β-subunit, SalI and HindIII sites were added at the 5′ and 3′ termini, respectively. The DNA fragments of double-alanine substitutions were inserted into the corresponding sites of pUCumA1CA2. Expression vectors for other double-alanine-substituted CumDO-Os (αK117A βR65A, αK117A βW100A, αK141A βR65A, and αK141A βW100A) were constructed as follows: The XbaI-SalI fragment containing a modified *cumA1* gene encoding αK117A or αK141A was cloned into the corresponding sites of pUCumA1CA2R65A or pUCumA1CA2W100A to replace wild-type genes with those with the appropriate mutations.

### Reduction efficiency analyses.

Upon receiving electrons, the Rieske cluster of CumDO-O is reduced, and the absorbance at 457 nm decreases (54). Based on the initial rate of decrease of absorbance at 457 nm, the efficiencies of electron transfer from CumDO-F to CumDO-O were quantified. The experimental conditions for single scans were as follows: bandwidth, 1.5 nm; wavelength, 457 nm; time, 80 s; temperature, 303 K. The protein solutions were dialyzed in TG buffer and diluted to 10 μM for CumDO-O and 0.2 μM for CumDO-R and CumDO-F. The absorbance spectra were collected on a Jasco V-630 spectrophotometer as follows. A total of 1,992 μL of TG buffer and a stir bar were added to a glass cuvette, which was sealed with rubber and aluminum caps. Oxygen was removed by bubbling with argon gas for 5 min. CumDO-R, CumDO-F, and CumDO-O were injected into the cuvette using a syringe (Hamilton, NV). A 100 mM concentration of NADH was added after measurement started. The slope of the initial rate was defined as the reduction efficiency. Measurements were performed three times independently for each sample. The data were assessed using Student's *t* test with *P* values of <0.005 or <0.001 for alanine-substituted CumDO-Os compared to the WT.

### Circular dichroism spectrum measurement.

Circular dichroism (CD) measurements were carried out using a Jasco J-820 spectropolarimeter with a 0.1-cm-path-length cuvette at room temperature under constant nitrogen gas flow. Samples were prepared at 0.3 mg/mL in 10 mM phosphate buffer (1.34 g/L Na_2_HPO_4_ [Wako Chemicals, Osaka, Japan], adjusted to pH 7.5 with NaH_2_PO_4_ [Wako Chemicals]). CD spectra were recorded from 190 to 260 nm at an interval of 0.2 nm and a scan speed of 200 nm/min. Each spectrum was the average of four successive scans.

### *In silico* analyses. (i) Homology modeling.

The structures of CumDO-F were modeled using SWISS-MODEL (32–36) based on the Rieske-type Fds in the 2.4-Å resolution crystal structure of the TDO-F–TDO-R complex of P. putida F1 (PDB entry 4EMJ) (37) or the 1.6-Å-resolution crystal structure of the BDO-F of Paraburkholderia xenovorans LB400 (PDB entry 1FQT) (8).

### (ii) Docking simulations.

Docking simulations were first performed using the global range molecular matching (GRAMM)-X protein-protein docking web server v.1.2.0 (55, 56). The crystal structure of the CumDO-O hexamer form (PDB entry 1WQL) (22) was used as the receptor protein and modeled CumDO-F as the ligand protein. The 20 best docking modes were analyzed. The docking modes showing distances between Rieske clusters of CumDO-F and CumDO-O of <14 Å (38) were considered the best-fit modes.

To analyze the detailed binding manner between CumDO-O and CumDO-F, complex structure modeling was performed as follows. The structure of the α_3_β_3_ biological assembly in CumDO-O was restored by using the BIOMT matrix described in the PDB file. Modeled structure using the crystal structure of BDO-F (PDB entry 1FQT) as described above was used as the tertiary structure of the CumDO-F. A complex structure between CumDO-O and CumDO-F that satisfied the following conditions was manually modeled by using UCSF Chimera (57): His45 or His66 of CumDO-F forms a hydrogen bond with an atom of CumDO-O that can mediate the electron transfer to the Rieske cluster of CumDO-O. Lys117 of the α-subunit and Arg65 of the β-subunit of CumDO-O can form salt bridges with acidic residues of the ferredoxin. Shape complementarity between the two proteins was also considered.

### (iii) Alignment analyses.

Multiple alignments were performed using ClustalX v.1.81 (58). Default settings were kept for all parameters except for matrix, which was BLOSUM for both pairwise and multiple alignments.

## References

[1] Kanaly RA, Harayama S. 2000. Biodegradation of high-molecular-weight polycyclic aromatic hydrocarbons by bacteria. J Bacteriol 182:2059–2067. 10.1128/JB.182.8.2059-2067.2000. 10735846 PMC111252

[2] Cerniglia CE. 1993. Biodegradation of polycyclic aromatic hydrocarbons. Curr Opin Cell Biol 4:331–338. 10.1016/0958-1669(93)90104-5.

[3] Gibson DT, Subramanian Z. 1984. Microbial degradation of aromatic compounds, p 181–252. *In* Gibson DT (ed), Microbial degradation of organic compounds. Marcel Dekker, New York, NY.

[4] Gibson DT, Parales RE. 2000. Aromatic hydrocarbon dioxygenases in environmental biotechnology. Curr Opin Biotechnol 11:236–243. 10.1016/s0958-1669(00)00090-2. 10851146

[5] Fuchs G, Boll M, Heider J. 2011. Microbial degradation of aromatic compounds—from one strategy to four. Nat Rev Microbiol 9:803–816. 10.1038/nrmicro2652. 21963803

[6] Ferraro DJ, Gakhar L, Ramaswamy S. 2005. Rieske business: structure-function of Rieske non-heme oxygenases. Biochem Biophys Res Commun 338:175–190. 10.1016/j.bbrc.2005.08.222. 16168954

[7] Wackett LP. 2002. Mechanism and applications of Rieske non-heme iron dioxygenases. Enzyme Microb Technol 31:577–587. 10.1016/S0141-0229(02)00129-1.

[8] Colbert CL, Couture MMJ, Eltis LD, Bolin JT. 2000. A cluster exposed: structure of the Rieske ferredoxin from biphenyl dioxygenase and the redox properties of Rieske Fe-S protein. Structure 8:1267–1278. 11188691 10.1016/s0969-2126(00)00536-0

[9] Batie CJ, Ballou DP, Correll CC. 1992. Phthalate dioxygenase reductase and related flavin-iron-sulfur containing electron transferases, p 543–556. *In* Muller F (ed), Chemistry and biochemistry of flavoenzymes, vol 3. CRC Press, Boca Raton, FL.

[10] Parales JV, Parales RE, Resnick SM, Gibson DT. 1998. Enzyme specificity of 2-nitrotoluene 2,3-dioxygenase from *Pseudomonas* sp. strain JS42 is determined by the C-terminal region of the β subunit of the oxygenase component. J Bacteriol 180:1194–1199. 10.1128/JB.180.5.1194-1199.1998. 9495758 PMC107007

[11] Kauppi B, Lee K, Carredano E, Parales RE, Gibson DT, Eklund H, Ramaswamy S. 1998. Structure of an aromatic-ring-hydroxylating dioxygenase—naphthalene 1,2-dioxygenase. Structure 6:571–586. 10.1016/s0969-2126(98)00059-8. 9634695

[12] Parales RE, Emig MD, Lynch NA, Gibson DT. 1998. Substrate specificities of hybrid naphthalene and 2,4-dinitrotoluene dioxygenase enzyme systems. J Bacteriol 180:2337–2344. 10.1128/JB.180.9.2337-2344.1998. 9573183 PMC107173

[13] Beil S, Mason JR, Timmis KN, Pieper DH. 1998. Identification of chlorobenzene dioxygenase sequence elements involved in dechlorination of identification of chlorobenzene dioxygenase sequence elements involved in dechlorination of 1,2,4,5-tetrachlorobenzene. J Bacteriol 180:5520–5528. 10.1128/JB.180.21.5520-5528.1998. 9791099 PMC107608

[14] Tan H-M, Cheong C-M. 1994. Substitution of the ISP α-subunit of biphenyl dioxygenase from *Pseudomonas* results in a modification of the enzyme activity. Biochem Biophys Res Commun 204:912–917. 10.1006/bbrc.1994.2546. 7980560

[15] Nojiri H, Ashikawa Y, Noguchi H, Nam J-W, Urata M, Fujimoto Z, Uchimura H, Terada T, Nakamura S, Shimizu K, Yoshida T, Habe H, Omori T. 2005. Structure of the terminal oxygenase component of angular dioxygenase, carbazole 1,9a-dioxygenase. J Mol Biol 351:355–370. 10.1016/j.jmb.2005.05.059. 16005887

[16] Senda M, Kishigami S, Kimura S, Fukuda M, Ishida T, Senda T. 2007. Molecular mechanism of the redox-dependent interaction between NADH-dependent ferredoxin reductase and Rieske-type [2Fe-2S] ferredoxin. J Mol Biol 373:382–400. 10.1016/j.jmb.2007.08.002. 17850818

[17] Friemann R, Lee K, Brown EN, Gibson DT, Eklund H, Ramaswamy S. 2009. Structures of the multicomponent Rieske non-heme iron toluene 2,3-dioxygenase enzyme system. Acta Crystallogr D Biol Crystallogr 65:24–33. 10.1107/S0907444908036524. 19153463 PMC2628974

[18] Ashikawa Y, Fujimoto Z, Noguchi H, Habe H, Omori T, Yamane H, Nojiri H. 2006. Electron transfer complex formation between oxygenase and ferredoxin components in Rieske nonheme iron oxygenase system. Structure 14:1779–1789. 10.1016/j.str.2006.10.004. 17161368

[19] Khara P, Roy M, Chakraborty J, Ghosal D, Dutta TK. 2014. Functional characterization of diverse ring-hydroxylating oxygenases and induction of complex aromatic catabolic gene clusters in *Sphingobium* sp. PNB. FEBS Open Bio 4:290–300. 10.1016/j.fob.2014.03.001. PMC4048848 24918041

[20] Kumari A, Singh D, Ramaswamy S, Ramanathan G. 2017. Structural and functional studies of ferredoxin and oxygenase components of 3-nitrotoluene dioxygenase from *Diaphorobacter* sp. strain DS2. PLoS One 12:e0176398. 10.1371/journal.pone.0176398. 28448625 PMC5407579

[21] Aoki H, Kimura T, Habe H, Yamane H, Kodama T, Omori T. 1996. Cloning, nucleotide sequence, and characterization of the genes encoding enzymes involved in the degradation of cumene to 2-hydroxy-6-oxo-7-methylocta-2,4-dienoic acid in *Pseudomonas fluorescens* IP01. J Ferment Bioeng 81:187–196. 10.1016/0922-338X(96)82207-0.

[22] Dong X, Fushinobu S, Fukuda E, Terada T, Nakamura S, Shimizu K, Nojiri H, Omori T, Shoun H, Wakagi T. 2005. Crystal structure of the terminal oxygenase component of cumene dioxygenase from *Pseudomonas fluorescens* IP01. J Bacteriol 187:2483–2490. 10.1128/JB.187.7.2483-2490.2005. 15774891 PMC1065230

[23] Furukawa K. 2000. Engineering dioxygenases for efficient degradation of environmental pollutants. Curr Opin Biotechnol 11:244–249. 10.1016/s0958-1669(00)00091-4. 10851151

[24] Furusawa Y, Nagarajan V, Tanokura M, Masai E, Fukuda M, Senda T. 2004. Crystal structure of the terminal oxygenase component of biphenyl dioxygenase derived from *Rhodococcus* sp. strain RHA1. J Mol Biol 342:1041–1052. 10.1016/j.jmb.2004.07.062. 15342255

[25] Suenaga H, Mitsuoka M, Ura Y, Watanabe T, Furukawa K. 2001. Directed evolution of biphenyl dioxygenase: emergence of enhanced degradation capacity for benzene, toluene, and alkylbenzenes. J Bacteriol 183:5441–5444. 10.1128/JB.183.18.5441-5444.2001. 11514531 PMC95430

[26] Suenaga H, Watanabe T, Sato M, Furukawa K. 2002. Alteration of regiospecificity in biphenyl dioxygenase by active-site engineering. J Bacteriol 184:3682–3688. 10.1128/JB.184.13.3682-3688.2002. 12057964 PMC135152

[27] Zielinski M, Backhaus S, Hofer B. 2002. The principal determinants for the structure of the substrate-binding pocket are located within a central core of a biphenyl dioxygenase α subunit. Microbiology (Reading) 148:2439–2448. 10.1099/00221287-148-8-2439. 12177337

[28] Zielinski M, Kahl S, Hecht HJ, Hofer B. 2003. Pinpointing biphenyl dioxygenase residues that are crucial for substrate interaction. J Bacteriol 185:6976–6980. 10.1128/JB.185.23.6976-6980.2003. 14617661 PMC262696

[29] Kumar P, Mohammadi M, Viger JF, Barriault D, Gomez-Gil L, Eltis LD, Bolin JT, Sylvestre M. 2011. Structural insight into the expanded PCB-degrading abilities of a biphenyl dioxygenase obtained by directed evolution. J Mol Biol 405:531–547. 10.1016/j.jmb.2010.11.009. 21073881 PMC3102011

[30] Werlen C, Kohler H-PE, van der Meer JR. 1996. The broad substrate chlorobenzene dioxygenase and *cis*-chlorobenzene dihydrodiol dehydrogenase of *Pseudomonas* sp. strain P51 are linked evolutionarily to the enzymes for benzene and toluene degradation. J Biol Chem 271:4009–4016. 10.1074/jbc.271.8.4009. 8626733

[31] Chakraborty J, Ghosal D, Dutta A, Dutta TK. 2012. An insight into the origin and functional evolution of bacterial aromatic ring-hydroxylating oxygenases. J Biomol Struct Dyn 30:419–436. 10.1080/07391102.2012.682208. 22694139

[32] Guex N, Peitsch MC, Schwede T. 2009. Automated comparative protein structure modeling with SWISS-MODEL and Swiss-PdbViewer: a historical perspective. Electrophoresis 30:S162–S173. 10.1002/elps.200900140. 19517507

[33] Benkert P, Biasini M, Schwede T. 2011. Toward the estimation of the absolute quality of individual protein structure models. Bioinformatics 27:343–350. 10.1093/bioinformatics/btq662. 21134891 PMC3031035

[34] Bertoni M, Kiefer F, Biasini M, Bordoli L, Schwede T. 2017. Modeling protein quaternary structure of homo- and hetero-oligomers beyond binary interactions by homology. Sci Rep 7:10480. 10.1038/s41598-017-09654-8. 28874689 PMC5585393

[35] Bienert S, Waterhouse A, de Beer TAP, Tauriello G, Studer G, Bordoli L, Schwede T. 2017. The SWISS-MODEL Repository—new features and functionality. Nucleic Acids Res 45:D313–D319. 10.1093/nar/gkw1132. 27899672 PMC5210589

[36] Waterhouse A, Bertoni M, Bienert S, Studer G, Tauriello G, Gumienny R, Heer FT, de Beer TAP, Rempfer C, Bordoli L, Lepore R, Schwede T. 2018. SWISS-MODEL: homology modelling of protein structures and complexes. Nucleic Acids Res 46:W296–W303. 10.1093/nar/gky427. 29788355 PMC6030848

[37] Lin T-Y, Werther T, Jeoung J-H, Dobbek H. 2012. Suppression of electron transfer to dioxygen by charge transfer and electron transfer complexes in the FAD-dependent reductase component of toluene dioxygenase. J Biol Chem 287:38338–38346. 10.1074/jbc.M112.374918. 22992736 PMC3488102

[38] Page CC, Moser CC, Chen X, Dutton PL. 1999. Natural engineering principles of electron tunneling in biological oxidation-reduction. Nature 402:47–52. 10.1038/46972. 10573417

[39] Colbert CL, Agar NYR, Kumar P, Chakko MN, Sinha SC, Powlowski JB, Eltis LD, Bolin JT. 2013. Structural characterization of *Pandoraea pnomenusa* B-356 biphenyl dioxygenase reveals features of potent polychlorinated biphenyl-degrading enzymes. PLoS One 8:e52550. 10.1371/journal.pone.0052550. 23308114 PMC3536784

[40] Ferraro DJ, Brown EN, Yu CL, Parales RE, Gibson DT, Ramaswamy S. 2007. Structural investigations of the ferredoxin and terminal oxygenase components of the biphenyl 2,3-dioxygenase from *Sphingobium yanoikuyae* B1. BMC Struct Biol 7:10. 10.1186/1472-6807-7-10. 17349044 PMC1847435

[41] Friemann R, Ivkovic-Jensen MM, Lessner DJ, Yu C-L, Gibson DT, Parales RE, Eklund H, Ramaswamy S. 2005. Structural insight into the dioxygenation of nitroarene compounds: the crystal structure of nitrobenzene dioxygenase. J Mol Biol 348:1139–1151. 10.1016/j.jmb.2005.03.052. 15854650

[42] Jakoncic J, Jouanneau Y, Meyer C, Stojanoff V. 2007. The crystal structure of the ring-hydroxylating dioxygenase from *Sphingomonas* CHY-1. FEBS J 274:2470–2481. 10.1111/j.1742-4658.2007.05783.x. 17451434

[43] Brown EN, Friemann R, Karlsson A, Parales JV, Couture MM, Eltis LD, Ramaswamy S. 2008. Determining Rieske cluster reduction potentials. J Biol Inorg Chem 13:1301–1313. 10.1007/s00775-008-0413-4. 18719951

[44] Nam J-W, Noguchi H, Fujimoto Z, Mizuno H, Ashikawa Y, Abo M, Fushinobu S, Kobashi N, Wakagi T, Iwata K, Yoshida T, Habe H, Yamane H, Omori T, Nojiri H. 2005. Crystal structure of the ferredoxin component of carbazole 1,9a-dioxygenase of *Pseudomonas resinovorans* strain CA10, a novel Rieske non-heme iron oxygenase system. Proteins 58:779–789. 10.1002/prot.20374. 15645447

[45] Inoue K, Ashikawa Y, Umeda T, Abo M, Katsuki J, Usami Y, Noguchi H, Fujimoto Z, Terada T, Yamane H, Nojiri H. 2009. Specific interactions between the ferredoxin and terminal oxygenase components of a class IIB Rieske nonheme iron oxygenase, carbazole 1,9a-dioxygenase. J Mol Biol 392:436–451. 10.1016/j.jmb.2009.07.029. 19616558

[46] Harayama S, Rekik M, Timmis KN. 1986. Genetic analysis of a relaxed substrate specificity aromatic ring dioxygenase, toluate 1,2-dioxygenase, encoded by TOL plasmid pWW0 of *Pseudomonas putida*. Mol Gen Genet 202:226–234. 10.1007/BF00331641. 3010045

[47] Hurtubise Y, Barriault D, Sylvestre M. 1998. Involvement of the terminal oxygenase β subunit in the biphenyl dioxygenase reactivity pattern toward chlorobiphenyls. J Bacteriol 180:5828–5835. 10.1128/JB.180.22.5828-5835.1998. 9811638 PMC107654

[48] Jiang H, Parales RE, Gibson DT. 1999. The α subunit of toluene dioxygenase from *Pseudomonas putida* F1 can accept electrons from reduced ferredoxin_TOL_ but is catalytically inactive in the absence of the β subunit. Appl Environ Microbiol 65:315–318. 10.1128/AEM.65.1.315-318.1999. 9872799 PMC91022

[49] Martins BM, Svetlitchnaia T, Dobbek H. 2005. 2-Oxoquinoline 8-monooxygenase oxygenase component: active site modulation by Rieske-[2Fe–2S] center oxidation/reduction. Structure 13:817–824. 10.1016/j.str.2005.03.008. 15893671

[50] Annalora AJ, Goodin DB, Hong W-X, Zhang Q, Johnson EF, Stout CD. 2010. Crystal structure of CYP24A1, a mitochondrial cytochrome P450 involved in vitamin D metabolism. J Mol Biol 396:441–451. 10.1016/j.jmb.2009.11.057. 19961857 PMC2830900

[51] Yang W, Bell SG, Wang H, Zhou W, Hoskins N, Dale A, Bartlam M, Wong L-L, Rao Z. 2010. Molecular characterization of a class I P450 electron transfer system from *Novosphingobium aromaticivorans* DSM12444. J Biol Chem 285:27372–27384. 10.1074/jbc.M110.118349. 20576606 PMC2930735

[52] Sambrook J, Russell D. 2001. Molecular cloning: a laboratory manual, 3rd ed. Cold Spring Harbor Laboratory Press, Cold Spring Harbor, NY.

[53] Nam J-W, Nojiri H, Noguchi H, Uchimura H, Yoshida T, Habe H, Yamane H, Omori T. 2002. Purification and characterization of carbazole 1,9a-dioxygenase, a three-component dioxygenase system of *Pseudomonas resinovorans* strain CA10. Appl Environ Microbiol 68:5882–5890. 10.1128/AEM.68.12.5882-5890.2002. 12450807 PMC134387

[54] Rosche B, Tshisuaka B, Fetzner S, Lingens F. 1995. 2-Oxo-1,2-dihydroquinoline 8-monooxygenase, a two-component enzyme system from *Pseudomonas putida* 86. J Biol Chem 270:17836–17842. 10.1074/jbc.270.30.17836. 7629085

[55] Tovchigrechko A, Vakser IA. 2005. Development and testing of an automated approach to protein docking. Proteins 60:296–301. 10.1002/prot.20573. 15981259

[56] Tovchigrechko A, Vakser IA. 2006. GRAMM-X public web server for protein-protein docking. Nucleic Acids Res 34:W310–W314. 10.1093/nar/gkl206. 16845016 PMC1538913

[57] Pettersen EF, Goddard TD, Huang CC, Couch GS, Greenblatt DM, Meng EC, Ferrin TE. 2004. UCSF Chimera—a visualization system for exploratory research and analysis. J Comput Chem 25:1605–1612. 10.1002/jcc.20084. 15264254

[58] Thompson JD, Gibson TJ, Plewniak F, Jeanmougin F, Higgins DG. 1997. The CLUSTAL_X windows interface: flexible strategies for multiple sequence alignment aided by quality analysis tools. Nucleic Acids Res 25:4876–4882. 10.1093/nar/25.24.4876. 9396791 PMC147148

