# Supplementary material for: The α- and β-Subunit Boundary at the Stem of the Mushroom-Like α3β3-Type Oxygenase Component of Rieske Non-Heme Iron Oxygenases Is the Rieske-Type Ferredoxin-Binding Site
Source: Appl Environ Microbiol. 2022 Jul 13;88(15):e00835-22. doi: 10.1128/aem.00835-22 (PMC9361823; doi:10.1128/aem.00835-22)
Supplement: Supplemental file 1 — Fig. S1 to S4 and Table S1. Download aem.00835-22-s0001.pdf, PDF file, 1.0 MB [file aem.00835-22-s0001.pdf]

Supplementary materials

The α- and β-Subunit Boundary at the Stem of the Mushroom-Like $\alpha_3\beta_3$-Type Oxygenase Component of Rieske Non-heme Iron Oxygenases is the Rieske-Type Ferredoxin-Binding Site

Pi-Cheng Tsai,[a] Joydeep Chakraborty,[a,b] Chiho Suzuki-Minakuchi,[a,c] Tohru Terada,[c,d] Tatsurou Kotake,[a] Jun Matsuzawa,[a] Kazunori Okada,[a] and Hideaki Nojiri[a,c]#

[a] Agro-Biotechnology Research Center, Graduate School of Agricultural and Life Sciences, The University of Tokyo, 1-1-1 Yayoi, Bunkyo-ku, Tokyo 113-8657, Japan

[b] Department of Microbiology, School of Science, RK University, Kasturbadham, Rajkot - 360020, Gujarat, India

[c] Collaborative Research Institute for Innovative Microbiology, The University of Tokyo, 1-1-1 Yayoi, Bunkyo-ku, Tokyo 113-8657, Japan

[d] Department of Biotechnology, Graduate School of Agricultural and Life Sciences, The University of Tokyo, 1-1-1 Yayoi, Bunkyo-ku, Tokyo, 113-8657, Japan

#Address correspondence to Hideaki NOJIRI, anojiri@g.ecc.u-tokyo.ac.jp

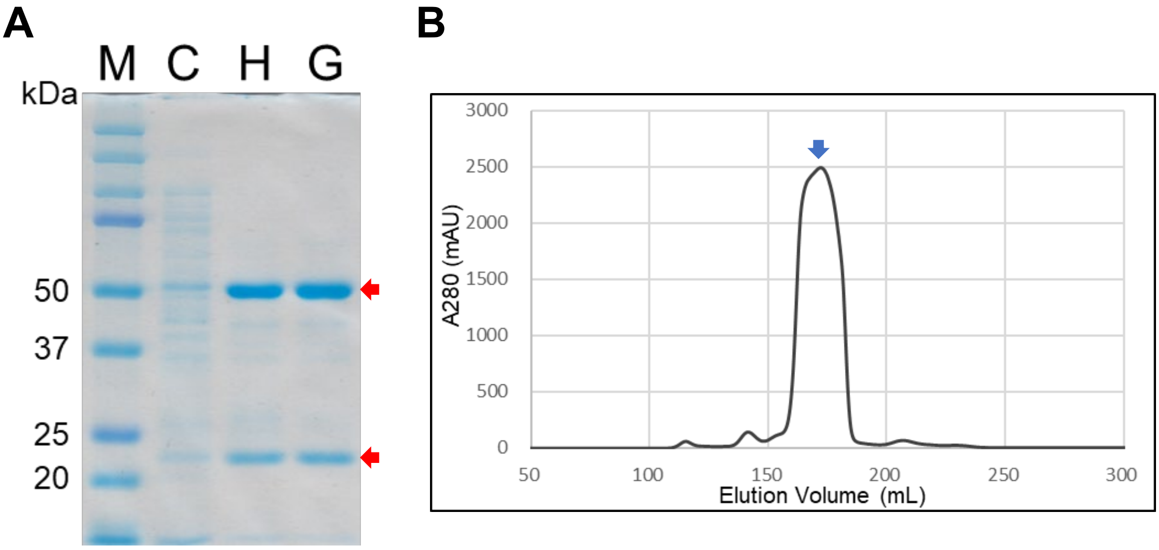

Figure S1. Elution profile of gel filtration chromatography of WT CDO-O

(A) 10% Glycine SDS-PAGE of WT CDO-O. Red arrows indicate α-subunit of CumDO-O (51 kDa) and β-subunit of CumDO-O (21 kDa). C: crude extract, H: purified by metal-chelation chromatography, G: purified by gel filtration chromatography, M: Precision Plus Protein™ Dual Color Standards. (B) Gel chromatography of the affinity purified WT CDO-O. Black line indicates absorbance at 280 nm (mAU). Blue arrow indicates the peak of CumDO-O.

A

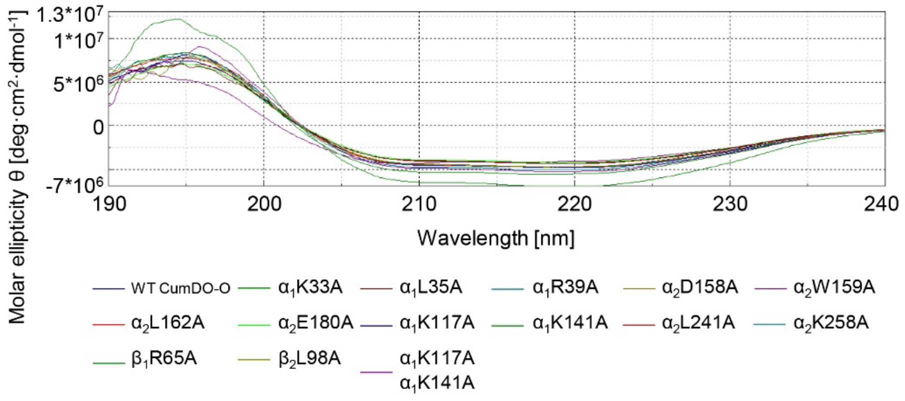

B

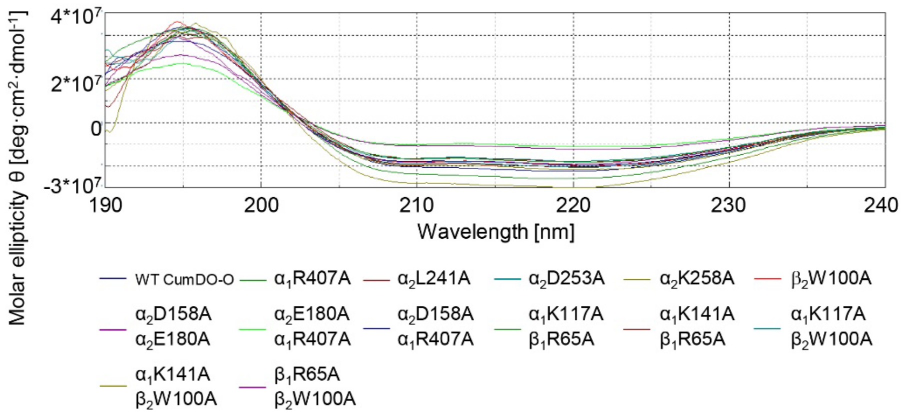

C

	wild-type	α_1K33A	α_1L35A	α_1R39A	α_1R407A	α_2D158A	α_2W159A	α_2L162A	α_2E180A
α-helix	35.60%	37.10%	38.50%	39.50%	39.80%	39.70%	30.50%	39.20%	38.10%
β-sheet	28.10%	27.30%	26.20%	24.50%	24.20%	26.80%	32.30%	24.20%	26.70%
turn	15.10%	16.10%	15.10%	16.20%	16.50%	14.20%	8.50%	16.10%	15.50%
random coil	21.10%	19.50%	20.10%	19.80%	19.50%	19.40%	28.80%	20.40%	19.70%

	α_1K117A	α_1K141A	α_2L241A	α_2D253A	α_2K258A	β_1R65A	β_2L98A	β_2W100A
α-helix	36.50%	32.70%	36.90%	34.40%	35.60%	35.90%	36.90%	37.90%
β-sheet	28.40%	31.10%	27.70%	30.70%	31.00%	31.20%	26.90%	25.20%
turn	15.30%	13.60%	15.60%	14.50%	14.20%	13.20%	15.40%	15.80%
random coil	19.80%	22.50%	19.80%	20.40%	19.10%	19.60%	20.80%	21.10%

	α_2D158Aα_2E180A	α_2D158Aα_1R407A	α_2E180Aα_1R407A	α_1K117Aα_1K141A	K117Aβ_1R65A	K117Aβ_2W100A	K141Aβ_2W100A	β_1R65Aβ_2W100A
α-helix	34.70%	36.90%	36.10%	38.70%	32.10%	35.50%	34.10%	32.90%
β-sheet	31.30%	29.80%	27.20%	25.00%	31.20%	27.80%	29.20%	32.10%
turn	11.10%	13.00%	14.10%	14.80%	16.90%	14.20%	15.10%	17.00%
random coil	22.90%	20.30%	22.60%	21.50%	19.80%	22.50%	21.60%	18.00%

Figure S2. Circular dichroic (CD) spectral profiles of alanine-substituted CumDO-Os.
(A) and (B) The CD spectral profiles measured for various samples in different days are shown in different panels. Protein concentrations of all samples were adjusted to 0.3 mg/mL. X-axis is the wavelength and Y-axis is the molar ellipticity θ. (C) Ratios of α-helix, β-sheet, turn and random coil in CumDO-O derivatives are shown.

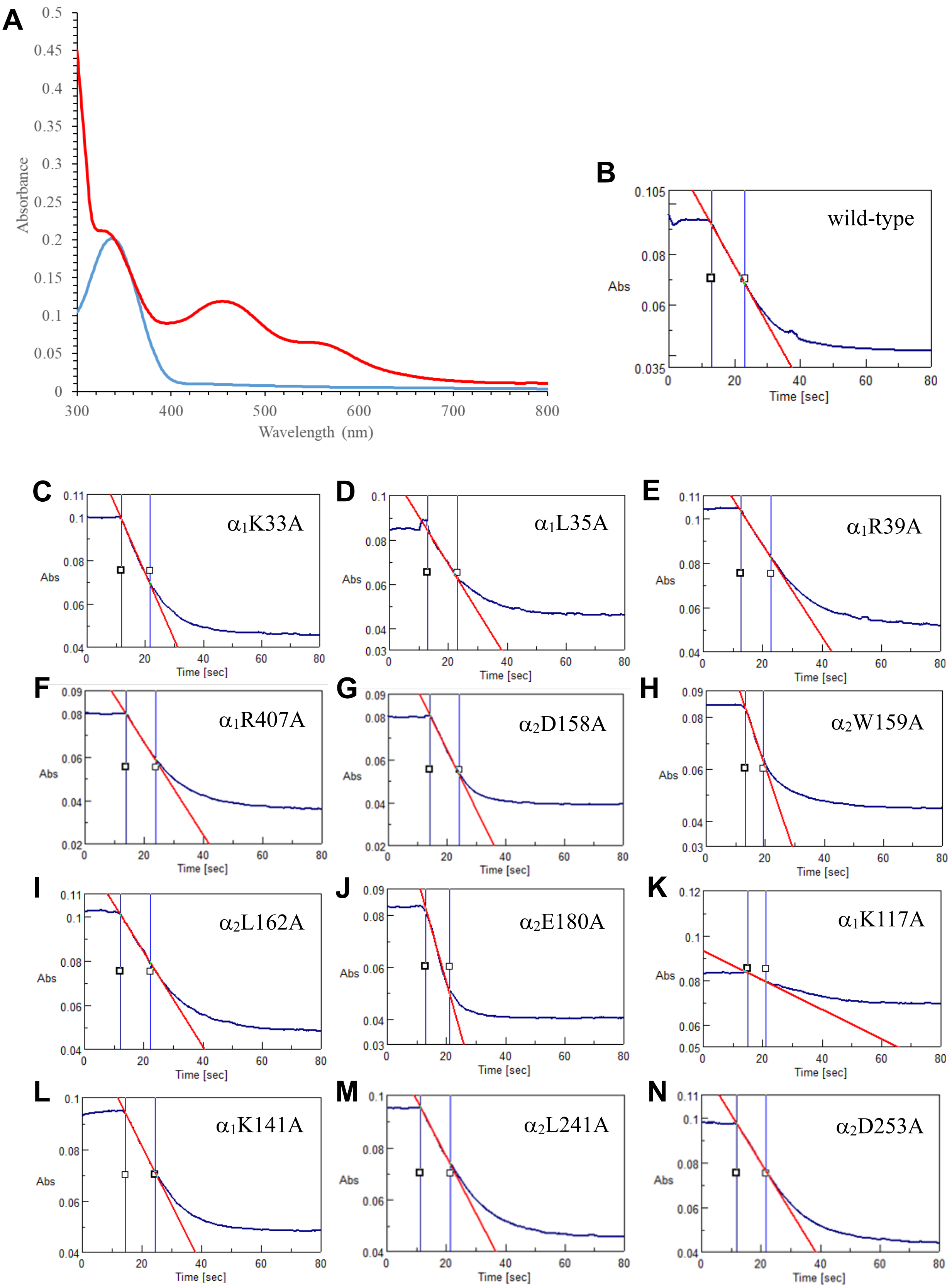
A
Absorbance
Wavelength (nm)
0.5
0.45
0.4
0.35
0.3
0.25
0.2
0.15
0.1
0.05
0
300
400
500
600
700
800
B
wild-type
Abs
Time [sec]
C
α1K33A
D
α1L35A
E
α1R39A
F
α1R407A
G
α2D158A
H
α2W159A
I
α2L162A
J
α2E180A
K
α1K117A
L
α1K141A
M
α2L241A
N
α2D253A

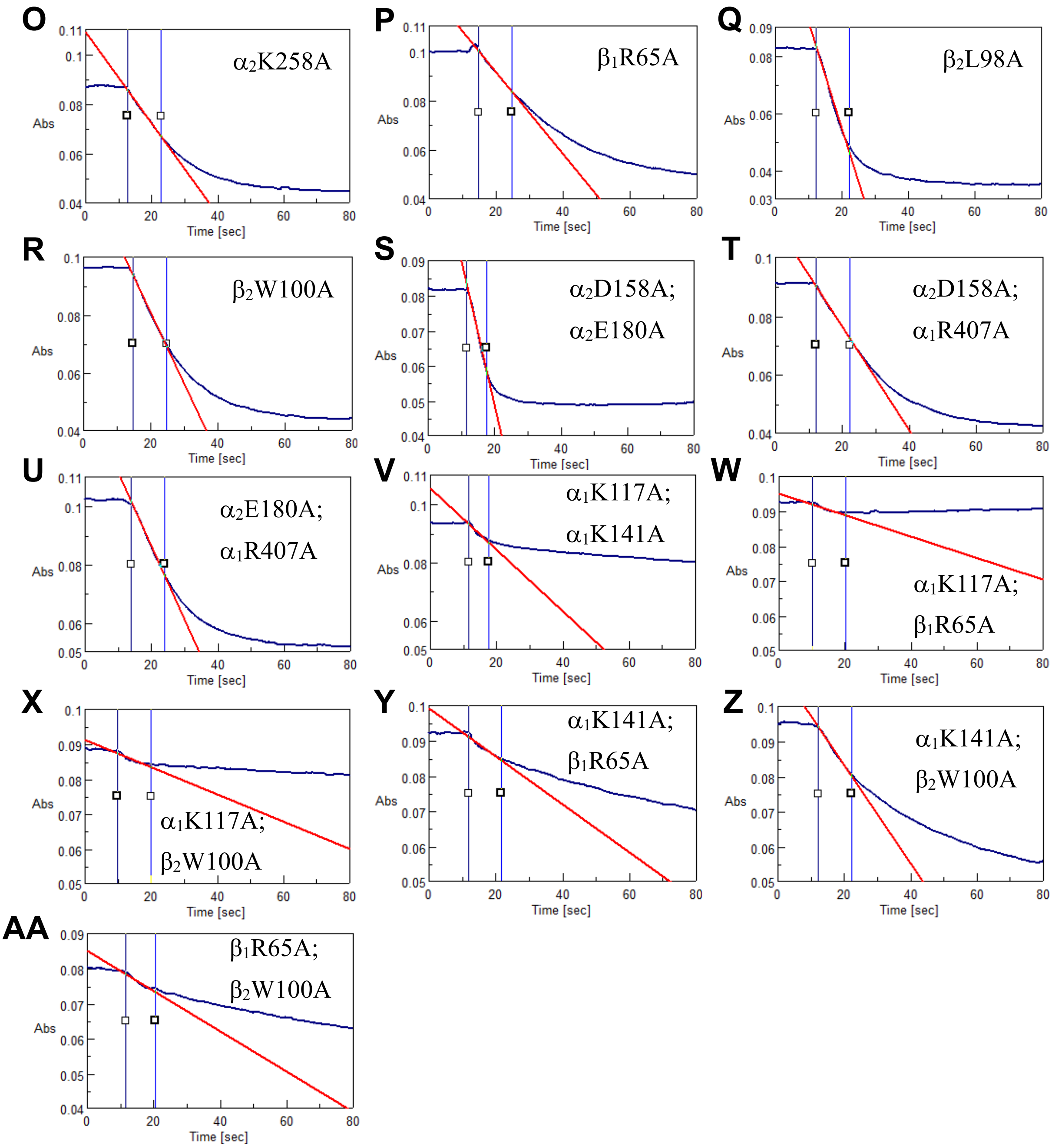

Figure S3. UV-Vis absorption spectrum and reduction efficiencies of WT CumDO-O and variants

(A) UV-Vis absorption spectrum of oxidized state (red) and reduced state (blue) WT CDO-Os. Oxidized state CumDO-O was the resting state after purification. To measure the spectrum for reduced state CumDO-O, absorbance spectra of mixture of excess amount of NADH, 0.2 μM

CumDO-R, 0.2 μM CumDO-F and 10 μM CumDO-O was recorded. The oxidized Rieske cluster has peak at 457 nm and peak shoulder at 570 nm. (B-D) The raw data of reduction efficiency of wild-type, α_1K33A, α_1L35A, α_1R39A, α_1R407A, α_2D158A, α_2W159A, α_2L162A, α_2E180A, α_1K117A, α_1K141A, α_2L241A, α_2D253A, α_2K258A, β_1R65A, β_2L98A, β_2W100A, α_2D158A;α_2E180A, α_2D158A;α_1R407A, α_2E180A;α_1R407A, α_1K117A;α_1K141A, α_1K117A;β_1R65A, α_1K117A;β_2W100A, α_1K141A;β_1R65A, α_1K141A;β_2W100A, and β_1R65A;β_2W100A (B-AA). The red line was fitted with the initial rate of absorbance decrease. The time range of calculation of red line was 10 seconds. We defined the slope of red line as reduction efficiency.

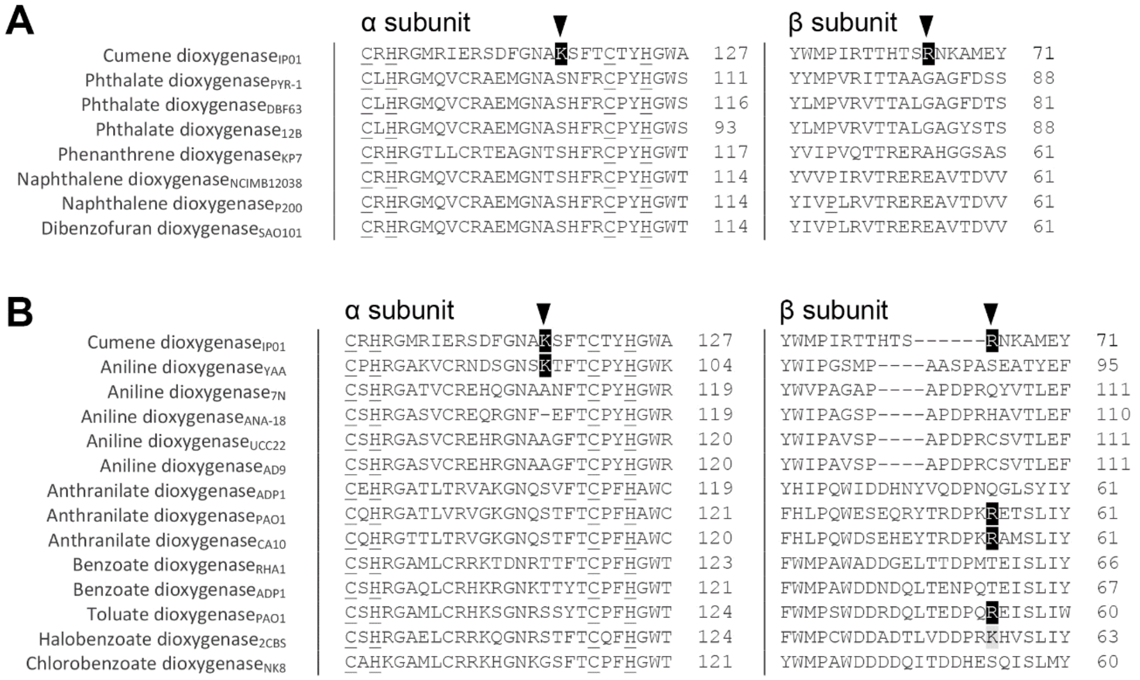

Figure S4. Amino acid sequence alignments of α- and β-subunits of CumDO Oxy with those of $\alpha_3\beta_3$-type Oxys coupled with non-Rieske-type Fds (A) and with Reds (B).

Only Oxy amino acid residues crucial for binding to Rieske-type Fds are shown. Positions of surface Lys and Arg residues proposed to be crucial for binding between $\alpha_3\beta_3$-type Oxys and Rieske-type Fds are indicated by arrow heads. At these positions, conserved and similar amino acid residues are dark- and light-shaded, respectively. The four conserved ligands for the Rieske cluster in α subunits are underlined. Numbers at right show the positions of terminal amino acid residues. Enzyme names and origins (subscripted) are shown at left.

Table S1. List of oxygenase components used in alignment analyses.

Name of RO [Organism]	α subunit		β subunit	
	Protein	Accession no.	Protein	Accession no.
Oxygenase components coupled with Rieske-type ferredoxin				
Cumene dioxygenase [*Pseudomonas fluorescens* IP01]	CumA1	BAA07074	CumA2	BAA07075
Isopropylbenzene dioxygenase [*Pseudomonas* sp. JR1]	IpbA1	AAB36666	IpbA2	AAB36667
Alkylbenzene dioxygenase [*Pseudomonas putida* 01G3]	EbdAa	CAB99196	EbdAb	CAB99197
Isopropylbenzene dioxygenase [*Pseudomonas putida* RE204]	IpbAa	AAC03436	IpbAb	AAC03437
Isopropylbenzene dioxygenase [*Rhodococcus erythropolis* BD2]	IpbA1	AAP74038	IpbA2	AAP74039
Alkylbenzene dioxygenase [*Rhodococcus* sp. DK17]	AkbA1a	AAR90139	AkbA2a	AAR90140
Biphenyl dioxygenase [*Dyella ginsengisoli* LA-4]	BphA1	ABX56038	BphA2	ABX56039
Biphenyl dioxygenase [*Pseudomonas furukawaii* KF707]	BphA1	AAA25743	BphA2	AAA25744
Biphenyl dioxygenase [*Achromobacter* sp. BP3]	BphA1	ACF20634	BphA2	ACF20635
Biphenyl dioxygenase [*Paraburkholderia xenovorans* LB400]	BphA	AAB63425	BphE	AAB63426
Biphenyl dioxygenase [*Pandoraea pnomenusa* B-356]	BphA	AAC44526	BphE	AAC44527
Biphenyl dioxygenase [*Comamonas testosteroni* TK102]	BphA1	BAC01052	BphA2	BAC01053
Biphenyl dioxygenase [*Cupriavidus oxalaticus* A5]	BphA1	CAD61140	BphA2	CAD61141
Biphenyl dioxygenase [*Lysobacter* sp. OC7]	BphA1	BAH80171	BphA2	BAH80172
Biphenyl dioxygenase [*Rhodococcus globerulus* P6]	BphA1	CAA56346	BphA2	CAA56347
Biphenyl dioxygenase [*Janibacter* sp. TYM3221]	BphAa	BAK52803	BphAb	BAK52804
Biphenyl dioxygenase [*Rhodococcus jostii* RHA1]	BphA1	BAA06868	BphA2	BAA06869
Toluene dioxygenase [*Pseudomonas putida* F1]	TodC1	AAA26005	TodC2	AAA26006
Benzene dioxygenase [*Pseudomonas putida* ML2]	BedC1	AAA17758	BedC2	AAA17759
Benzene dioxygenase [*Rhodococcus opacus* B4]	BnzA1	BAD95523	BnzA2	BAD95524
Chlorobenzene dioxygenase [*Burkholderia* sp. PS12]	TecA1	AAC46390	TecA2	AAC46391
Chlorobenzene dioxygenase [*Pseudomonas nitroreducens* J5-1]	TcbAa	ABR26225	TcbAb	ABR26226
Chlorobenzene dioxygenase [*Pseudomonas* sp. P51]	TcbAa	AAC43632	TcbAb	AAC43633
Dibenzofuran dioxygenase [*Novosphingobium* sp. KA1]	DbfA1	WP_011608136	DbfA2	WP_011608137

Carbazole dioxygenase [*Sphingomonas* sp. CB3]	CarAa	AAC38616	CarAb	AAC38617
Tetralin dioxygenase [*Sphingopyxis macrogoltabida* TFA]	ThnA1	AAN26443	ThnA2	AAN26444
Naphthalene dioxygenase [*Pseudomonas aeruginosa* PaK1]	PahA3	BAA12240	PahA4	BAA12241
Naphthalene dioxygenase [*Pseudomonas stutzeri* AN10]	NahAc	AAD02136	NahAd	AAD02137
Naphthalene dioxygenase [*Polaromonas naphthalenivorans* CJ2]	NagAc	AAZ93388	NagAd	AAZ93389
Naphthalene dioxygenase [*Ralstonia* sp. U2]	NagAc	AAD12610	NagAd	AAD12611
Naphthalene dioxygenase [*Pseudomonas putida* G7]	NahAc	AAA25902	NahAd	AAA25903
Naphthalene dioxygenase [*Pseudomonas putida* ND6]	NahAc	AAP44288	NahAd	AAP44289
Naphthalene dioxygenase [*Pseudomonas putida* BS202]	NahA3	AAB62707	NahA4	AAB62708
Naphthalene dioxygenase [*Pseudomonas putida* NCIB 9816-4]	NahAc	AAO64274	NahAd	AAO64275
Naphthalene dioxygenase [*Pseudomonas* sp. C18]	-	4HJL_A	-	4HJL_B
Nitrobenzene dioxygenase [*Comamonas* sp. JS765]	NbzAc	AAL76202	NbzAd	AAL76203
Nitrotoluene dioxygenase [*Pseudomonas* sp. JS42]	NtdAc	AAB40383	NtdAd	AAB40384
Dinitrotoluene dioxygenase [*Burkholderia cepacia* R34]	DntAc	AAL50021	DntAd	AAL50020
Dinitrotoluene dioxygenase [*Burkholderia* sp. RASC]	DntAc	AAB09766	DntAd	AAB09767
Phenanthrene dioxygenase [*Alcaligenes faecalis* AFK2]	PhnAc	BAA76323	PhnAd	BAA76324
PAH dioxygenase [*Pseudomonas putida* OUS82]	PahAc	BAA20391	PahAd	BAA20392
PAH dioxygenase [*Cycloclasticus* sp. A5]	PhnA1	BAC81541	PhnA2	BAC81542
Biphenyl dioxygenase [*Sphingobium yanoikuyae* B1]	BphA1f	ABM91740	BphA2f	ABM91741
PAH dioxygenase [*Sphingomonas* sp. CHY-1]	PhnA1a	CAG17576	PhnA1a	CAG17577
Oxygenase components coupled with non-Rieske ferredoxin				
Phthalate dioxygenase [*Mycolicibacterium vanbaalenii* PYR-1]	PhtAa	AAQ91914	PhtAb	AAQ91915
Phthalate dioxygenase [*Terrabacter* sp. DBF63]	PhtA1	BAC54156	PhtA2	BAC54157
Phthalate dioxygenase [*Arthrobacter keyseri* 12B]	PhtAa	AAK16534	PhtAb	AAK16535
Phenanthrene dioxygenase [*Nocardioides* sp. KP7]	PhdA	BAA94708	PhdB	BAA94709
Naphthalene dioxygenase [*Rhodococcus* sp. NCIMB 12038]	NarAa	AAD28100	NarAb	AAD28101

Naphthalene dioxygenase [*Rhodococcus* sp. P200]	NarAa	AAR05114	NarAb	AAR05115
Dibenzofuran dioxygenase [*Rhodococcus opacus* SAO 101]	DodA	BAD02377	DodB	BAD02378
ROs without ferredoxin component				
Aniline dioxygenase [*Acinetobacter* sp. YAA]	AtdA3	BAA13012	AtdA3	BAA13013
Aniline dioxygenase [*Delftia acidovorans* 7N]	Orf7NC	BAD61049	Orf7ND	BAD61050
Aniline dioxygenase [*Frateuria* sp. ANA-18]	TdnA1	BAC82526	TdnA2	BAC82527
Aniline dioxygenase [*Pseudomonas putida* UCC22]	TdnA1	BAA12807	TdnA1	BAA12808
Aniline dioxygenase [*Delftia tsuruhatensis* AD9]	TadA1	AAX47241	TadA1	AAX47242
Anthranilate dioxygenase [*Acinetobacter baylyi* ADP1]	AntA	AAC34813	AntB	AAC34814
Anthranilate dioxygenase [*Pseudomonas aeruginosa* PAO1]	AntA	AAG05900	AntB	AAG05901
Anthranilate dioxygenase [*Pseudomonas resinovorans* CA10]	AntA	BAB32747	AntB	BAB32746
Benzoate dioxygenase [*Rhodococcus jostii* RHA1]	BenA	BAB70698	BenB	BAB70699
Benzoate dioxygenase [*Acinetobacter baylyi* ADP1]	BenA	AAC46436	BenB	AAC46437
Toluate dioxygenase [*Pseudomonas aeruginosa* PAO1]	XylX	AAG05906	XylY	AAG05905
Halobenzoate dioxygenase [*Burkholderia cepacia* 2CBS]	CbdA	CAA55681	CbdB	CAA55682
Chlorobenzoate dioxygenase [*Burkholderia* sp. NK8]	CbeA	BAB21463	CbeB	BAB21464
